# *Drosophila* hemocytes recognize lymph gland tumors of *mxc* mutants and activate the innate immune pathway in a reactive oxygen species-dependent manner

**DOI:** 10.1242/bio.059523

**Published:** 2022-11-03

**Authors:** Suzuko Kinoshita, Kazuki Takarada, Yuriko Kinoshita, Yoshihiro H. Inoue

**Affiliations:** Biomedical Research Center, Kyoto Institute of Technology, Matsugasaki, Sakyo-ku, Kyoto, 606-8585, Japan

**Keywords:** *Drosophila*, Tumor, Hemocytes, ROS, Dual oxidase, Innate immunity

## Abstract

Mechanisms of cancer cell recognition and elimination by the innate immune system remains unclear. The immune signaling pathways are activated in the fat body to suppress the tumor growth in *mxc^mbn1^* hematopoietic tumor mutants in *Drosophila* by inducing antimicrobial peptides (AMP). Here, we investigated the regulatory mechanism underlying the activation in the mutant. Firstly, we found that reactive oxygen species (ROS) accumulated in the hemocytes due to induction of dual oxidase and one of its activators. This was required for the AMP induction and the tumor growth suppression. Next, more hemocytes transplanted from normal larvae were associated with the mutant tumor than normal lymph glands (LGs). Matrix metalloproteinase 1 and 2 (MMP2) were highly expressed in the tumors. The basement membrane components in the tumors were reduced and ultimately lost inside. Depletion of the MMP2 rather than MMP1 resulted in a significantly reduced AMP expression in the mutant larvae. The hemocytes may recognize the disassembly of basement membrane in the tumors and activate the ROS production. Our findings highlight the mechanism via which macrophage-like hemocytes recognize tumor cells and subsequently convey the information to induce AMPs in the fat body. They contribute to uncover the role of innate immune system against cancer.

## Teaser

**Summary:** *Drosophila* hemocytes recognized disintegrated basement membrane of the LG tumors overexpressing MMP2 and stimulated ROS production to induce the anti-tumor peptides under innate immune pathway in the fat body.

## INTRODUCTION

Insects rely fundamentally on the innate immune system to escape parasite-microbial infection, as they lack an acquired immune system (Brennan and Anderson, 2004; Buchon et al., 2014; Hoffmann, 2003). The innate immunity of *Drosophila* is roughly divided into two types, humoral and cell-mediated immune responses. Humoral immunity involves the use of anti-microbial peptides (AMPs), which are synthesized in the fat body and secreted into the hemolymph (Hoffmann and Reichhart, 2002; Tzou et al., 2002). Seven distinct AMPs, together with their isoforms, have been identified in *Drosophila melanogaster* (Lemaitre and Hoffmann, 2007; Martinelli and Reichhart, 2005). *Drosophila* AMPs can not only destroy invading microorganisms but can also suppress the progression of several types of tumors in the larvae (Araki et al., 2019; Bilder et al., 2021; Parvy et al., 2019). Araki and colleagues have reported that overexpression of any one of the five AMPs studied enhanced apoptosis in *mxc* tumor lymph glands (LGs), whereas no apoptotic signals were detected in the controls. Expression of these AMP genes is regulated by two major innate immune signaling pathways (Buchon et al., 2014; Hoffmann and Reichhart, 2002; Tzou et al., 2002). The first one is the Toll-mediated pathway, which is activated mainly by gram-positive bacteria and fungi (Lemaitre et al., 1997). Infection of these microbes initiates activation of consecutive serine protease cascades, which ultimately lead to the production of an active ligand called Spätzle (Jang et al., 2006). The active ligand binds and activates the transmembrane receptor, Toll, (Buchon et al., 2014; Morisato and Anderson, 1994). This stimulates phosphorylation and degradation of Cactus, the negative regulator of the NF-κB family of transcription factors Dorsal (Dl) and Dorsal-related immunity factor (Dif) (Sun et al., 2002). In response to infection, these transcription factors are free to translocate into the nucleus and induce the expression of genes encoding antimicrobial peptides such as Drosomycin (Drs) (Fehlbaum et al., 1994; Hoffmann, 2003). The second innate immune signaling pathway is activated by infection of Gram-negative bacteria. These bacteria are recognized by peptidoglycan recognition proteins (PGRP)-LC, LE and SD (Gottar et al., 2002), which leads to the activation of the signaling pathway mediated by a multiprotein complex containing Imd (Choe et al., 2005; Georgel et al., 2001; Lemaitre et al., 1995). Subsequently, the complex phosphorylates the Relish transcription factor (Rel), which triggers the cleavage of Rel (Kleino et al., 2005; Silverman et al., 2000). The resultant N terminal Rel drives the expression of several antimicrobial peptides, such as Diptericin (Dpt) (Stöven et al., 2003).

Cellular immune response also plays a significant role in protecting against invading pathogens in *Drosophila*. Three types of differentiated hemocytes are generated from blood cell progenitors called pro-hemocytes: plasmatocytes, lamellocytes, and crystal cells (Evans et al., 2003; Govind, 2008). The circulating hemocytes arise from two distinct hematopoietic tissues, the embryonic head mesoderm and a specialized organ called the LG at a later larval stage (Lanot et al., 2001; Lemaitre and Hoffmann, 2007; Araki et al., 2019; Morin-Poulard et al., 2021). In case of infection, phagocytosis by macrophage-like hemocytes called plasmatocytes and encapsulation and melanization by other two types of hemocytes come into play. *Drosophila* can respond to invading pathogenic microorganisms, as well as to tumor cells in the body (Wang et al., 2014). The hemocytes associate with the tumors and activate the innate immune system, thereby restricting tumor growth (Parisi et al., 2014; Pastor-Pareja et al., 2008; Wang et al., 2014). A series of studies have investigated the crosstalk between cellular immune response and tumors in *Drosophila* (Arefin et al., 2017; Kalamarz et al., 2012; Kim and Choe, 2014). Two AMPs, Drosomycin and Defensin, seemed to be incorporated by circulating hemocytes associated with the LG tumors (Araki et al., 2019). These results suggested that AMPs possess a specific cytotoxic property, because of which they induce apoptosis exclusively in the tumors. Another subsequent study demonstrated that Defensin can suppress tumor progression by targeting phosphatidyl serine on tumor cell membranes and inducing cell death cooperating with tumor necrosis factor in *Drosophila* imaginal discs (Parvy et al., 2019; Hanson and Lemaitre, 2020). However, the mechanism via which the humoral response is triggered in the response to activation of the cellular innate immune system that detected tumor cells still remain unknown. Furthermore, the mechanism via which the information is transmitted from the LG toward the fat body to induce AMPs in the tissue is not understood.

*Drosophila* harboring mutation in an allele of *multi sex combs* (*mxc*), *mxc^mxc^*, has been considered to be a unique hematopoietic tumor model (Remillieux-Leschelle et al., 2002; Shrestha and Gateff, 1982; Araki et al., 2019; Kurihara et al., 2020a,b). The *mxc^mbn1^* mutant, which harbors a loss of function mutation in *mxc* gene encoding a known protein required for DNA-replication-dependent expression of five canonical histones*,* in contrast that many leukemia and/or lymphoma involved in complex gene arrangement and/or expression of chimeric proteins. The mutant larvae exhibit hyperplasia of undifferentiated cells in the LG. The mutant LG cells proliferated further, invaded host tissues, and ultimately killed the host when implanted into normal adult abdomens (Remillieux-Leschelle et al., 2002; Kurihara et al., 2020a,b). Because of this, the mutant LG cells are considered malignant tumors (Kurihara et al., 2020a; Remillieux-Leschelle et al., 2002; Shrestha and Gateff, 1982). Araki and colleagues demonstrated that the innate immune pathways were activated in the mutant larvae (Araki et al., 2019). Therefore, this model has advantage in investigation of interaction between tumors and the immune system in *Drosophila*.

Using the *mxc^mbn1^* larval hematopoietic tumor model in this study, we aim to identify the mechanisms mediating LG tumors/immune system interactions, including how tumors are recognized by the immune system and consequently lead to activation of AMPs in the fat body. First, we found that reactive oxygen species (ROS) were induced in hemocytes in the mutant larvae. Thus, we investigated whether the ROS induction was required for the activation of the innate immune system and suppression of tumor growth. For the purpose, we inhibited the ROS accumulation in the mutant larvae by feeding of antioxidant and investigated whether it influenced AMP induction and the LG hyperplasia. We further investigated whether known factors required for the ROS induction were upregulated and whether they were essential for the activation of innate immune pathway. Next, we addressed the mechanism by which the LG tumors could be recognized by the immune system. Many studies have shown that altered proteolysis of the extracellular matrix (ECM) is related to unregulated tumor growth, tissue invasion, and metastasis. The MMPs are the most prominent proteinases associated with tumorigenesis (Uhlirova and Bohmann, 2006; Igaki et al., 2006; Cordero et al., 2010; Zhai et al., 2015). A previous study has also shown that the expression of the MMP1, which cleaves the ECM proteins, was upregulated in the *mxc^mbn1^* mutant larvae, and that the proteinase was required for LG hyperplasia. Hence, the authors speculated that reduction in ECM due to MMP1 upregulation influenced the tumor phenotype in the *mxc^mbn1^* larvae (Kurihara et al., 2020a,b). Here, we investigated whether the MMPs were upregulated in the *mxc* tumor LGs. We further showed that the amount of basement membrane component in the mutant LGs overexpressing *Mmp2* was reduced. Taking these findings together, we propose a model that circulating hemocytes are involved in recognition of the LG tumors and possibly in conveying the information toward the fat body. The findings in this study will improve our understanding of the mechanisms via which the innate immune system in the fat body is activated in response to the presence of tumors, and, consequently, how the AMPs were secreted from the immune tissues.

## RESULTS

### Inhibition of the ROS accumulation by antioxidant feeding reduced AMP induction and enhancement of LG hyperplasia in *mxc^mbn1^* larvae

**Fig. 1.**
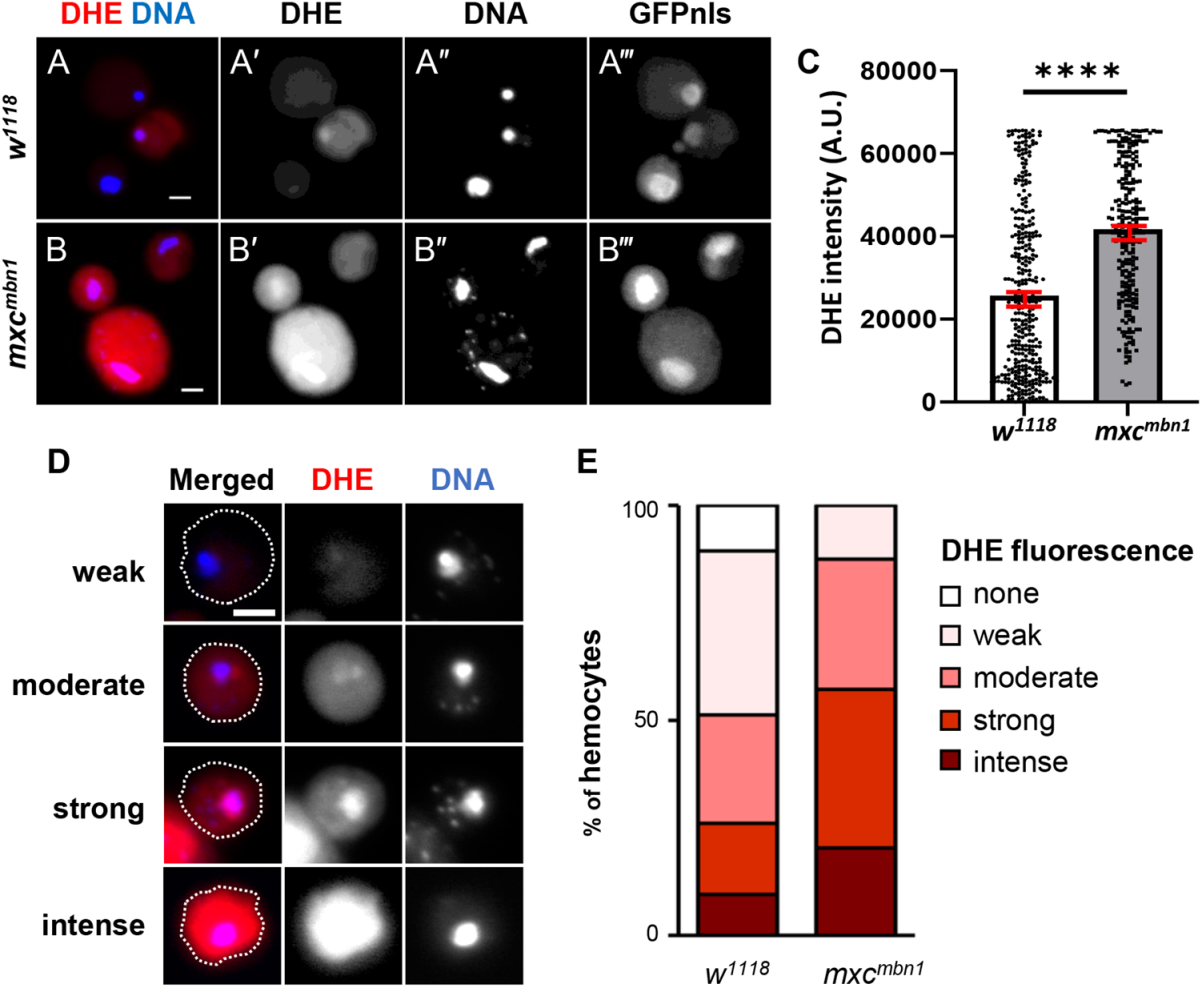
**Hyper-accumulation of ROS in circulating hemocytes of *mxc^mbn1^* mutant larvae.** (A,B) DHE staining of circulating hemocytes from matured third instar larva (A) normal control (*w/Y; He>GFPnls*), (B) *mxc^mbn1^* mutant (*mxc^mbn1^/Y; He>GFPnls*). DHE fluorescence and DNA staining by DAPI are colored in red (A, B, white in A′, B′) and in blue (A, B, white in A″, B″). A circulating hemocyte-specific GFP fluorescence (A‴, B‴). Scale bar: 5 μm. (C) Average arbitrary units of DHE fluorescence in circulating hemocytes (*n*≥247) from normal control (white bar) and *mxc^mbn1^* mutant larvae (gray bar), respectively. The distribution of each intensity is plotted on the bars. Error bards in red; standard error of mean (s.e.m.). (D) Typical images of the cells classified into the four classes according to the DHE fluorescence intensity (weak, moderate, strong, intense classes). The cell margins are encircled by dotted lines. Scale bar: 5 μm. (E) A percentage of each class of the DHE-stained circulating hemocytes from control (*w*) and *mxc^mbn1^* larvae. Among the hemocytes in the hemolymph of control larvae (*n*=247) 10.6% were in Class I, 38.1% in Class II, 25.2% in Class III, and 26.1% in Class IV. In contrast, 0.0% of the *mxc^mbn1^* hemocytes (*n*=357) were in Class I, 12.6% in Class II, 30.4% in Class III, and 57.0% in Class IV.

We first investigated whether ROS had accumulated in *mxc^mbn1^* larvae harboring the LG tumor. We performed dihydroethidium (DHE) staining of hemocytes obtained from the hemolymph of *mxc^mbn1^* larvae (*mxc^mbn1^/Y; He>GFPnls*) and of the cells from control larvae (*w/Y; He>GFPnls*) (Fig. 1A-C). We measured the DHE fluorescence in cells showing hemocyte-specific GFP fluorescence (Fig. 1A‴,B‴). DHE fluorescence increased significantly in *mxc^mbn1^* hemocytes (median intensity 43,116, *n*=247) (*P<*0.0001) compared to that in controls (median intensity value 21,100, *n*=357) (Fig. 1C). As the DHE fluorescence varied in intensity even among control hemocytes, we classified hemocytes into four classes [background level (Class I), weak (Class II), middle (Class III), and strong (Class IV)] according to the level of DHE fluorescence (Fig. 1D,E). These counting of each class indicated that ROS had accumulated in the hemocytes of *mxc^mbn1^* larvae. To confirm these results, we further investigated the fluorescence of the *gstD1-GFP* reporter, expression of which depends on ROS accumulation (Fig. S1). As expected, we observed significantly higher *gstD1* expression in the *mxc^mbn1^* hemocytes than in the controls (Fig. S1A'-D′,E), and the proportion of hemocytes showing stronger GFP fluorescence was higher in *mxc^mbn1^* than that in the controls (Fig. S1F,G). Next, we examined whether ROS also accumulated in *mxc^mbn1^* larval LGs where hemocyte precursors were produced. Using the *gstD1-GFP* reporter, we compared the ROS levels in the LGs (Fig. S2A-C). Remarkably, higher ROS levels were not observed in the *mxc* tumor LGs (only 40% increase), compared to that in the controls (Fig. S2A′, S2B′, and S2C), except that there were hemocyte-looking cells displaying more intense GFP fluorescence on the LGs (Fig. S2B, S2B′).

To understand the significance of ROS accumulation, we next investigated whether inhibition of the accumulation influenced the innate immune pathway and LG hyperplasia. To eliminate the ROS, we administered the N-acetyl cysteine (NAC)-supplemented diet to control larvae for 6 days until pupariation, and to *mxc^mbn1^* larvae for 11 days. The level of *gstD-GFP* expression in hemocytes of control larvae (median intensity 33.46, *n*=466) did not change distinctly, compared to that in the larvae not fed NAC (median intensity 30.46, *n*=372) (*P<*0.001, Fig. S1A,B,E). In contrast, the fluorescence in the mutant hemocytes decreased significantly after NAC feeding (median 33.49, *n*=259) compared to that in the mutant larvae without NAC feeding (median 70.54, *n*=174) (*P<*0.0001, Fig. S1C-E). Similarly, we classified the hemocytes after NAC feeding into the four classes on the basis of GFP fluorescence (Fig. S1F,G). The NAC feeding reduced ROS accumulation in hemocytes of *mxc^mbn1^*, but not in control larvae (Fig. S1G).

**Fig. 2.**
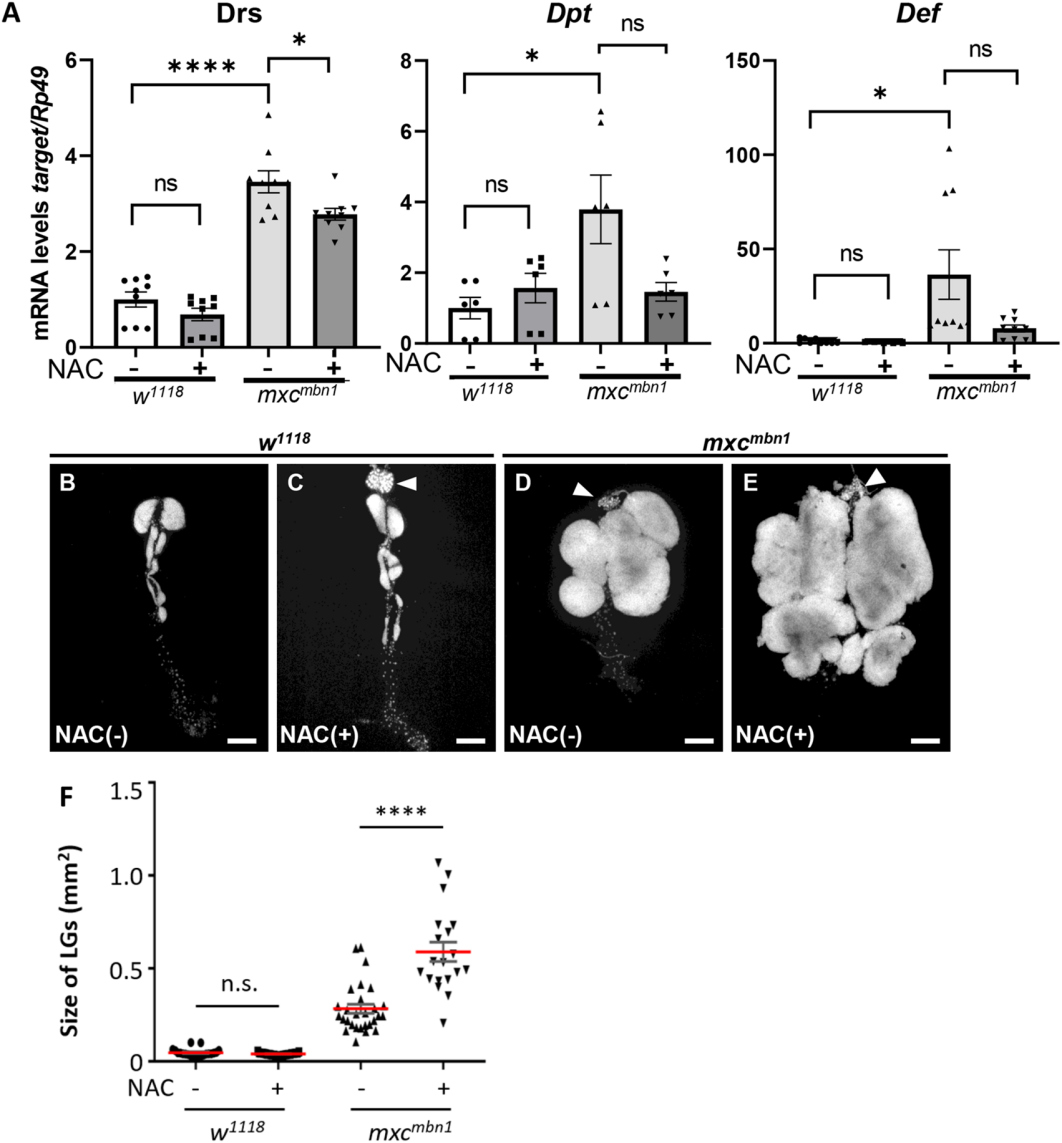
**Reduced levels of AMPs and enlarged LG tumors in *mxc^mbn1^* larvae fed on NAC.** (A) The mRNA levels of *Drs, Def,* and *Dpt* in fat bodies quantified by qRT-PCR. Total RNA was prepared from control male larvae (*w^1118^/Y*) fed without NAC, with NAC, the mutant male larvae (*mxc^mbn1^/Y*) fed without, or with NAC. The average level in normal control (*w^1118^*) is presented as 1.0. Error bar: s.e.m. (B-E) The DAPI-stained LGs from matured third instar larvae of control (*w^1118^/Y*) fed without NAC (B), with NAC (C), the mutant larvae (*mxc^mbn1^/Y*) fed without NAC (D), with NAC (E). Arrowhead indicates the ring gland linked to the LG. Scale bar: 200 µm. (F) Average size of the LGs from third instar larvae at mature stage (*n*≥19). The red lines indicate average size of the LG size. For statistical analysis of the differences between larvae with or without NAC feeding, Kruskal–Wallis test followed by the Mann–Whitney *U* test using Bonferroni correction was performed.

Next, we investigated whether the reduction in ROS level due to NAC feeding influenced activation of innate signaling in the fat body. The mRNA levels of *Drs* and *Def* in control larvae fed the NAC-containing diet decreased by 68.7% and 48.9% compared to the levels in non-fed larvae, respectively. The mRNA level of *Dpt* increased by 50%. In contrast, in *mxc^mbn1^* larvae, the average mRNA levels of all three genes in the fat body decreased by 81.5%, 38.3%, and 21.7%, respectively (Fig. 2A). Activation of the innate immune pathway in *mxc^mbn1^* larvae was inhibited with reduction in ROS levels. Moreover, we investigated whether reduction in ROS levels affected LG hyperplasia in *mxc^mbn1^* larvae (Fig. 2B-F). LG size did not differ significantly between control larvae fed NAC diet (median 0.04 mm^2^, *n*=21) and those fed diet without NAC (median 0.04 mm^2^, *n*=29) (Fig. 2F). However, the LG hyperplasia in *mxc^mbn1^* larvae (median 0.53 mm^2^, *n*=19) was significantly higher than that in the mutant larvae not fed the NAC diet (median 0.25 mm^2^, *n*=28) (*P<*0.0001) (Fig. 2F). We concluded that ROS accumulation in *mxc^mbn1^* larvae was required for the activation of the innate signaling pathway in the fat body, resulting in suppression of LG hyperplasia.

### *Duox* was upregulated in *mxc^mbn1^* larvae, and its depletion in hemocytes reduced the mRNA levels of AMP genes and increased LG hyperplasia

**Fig. 3.**
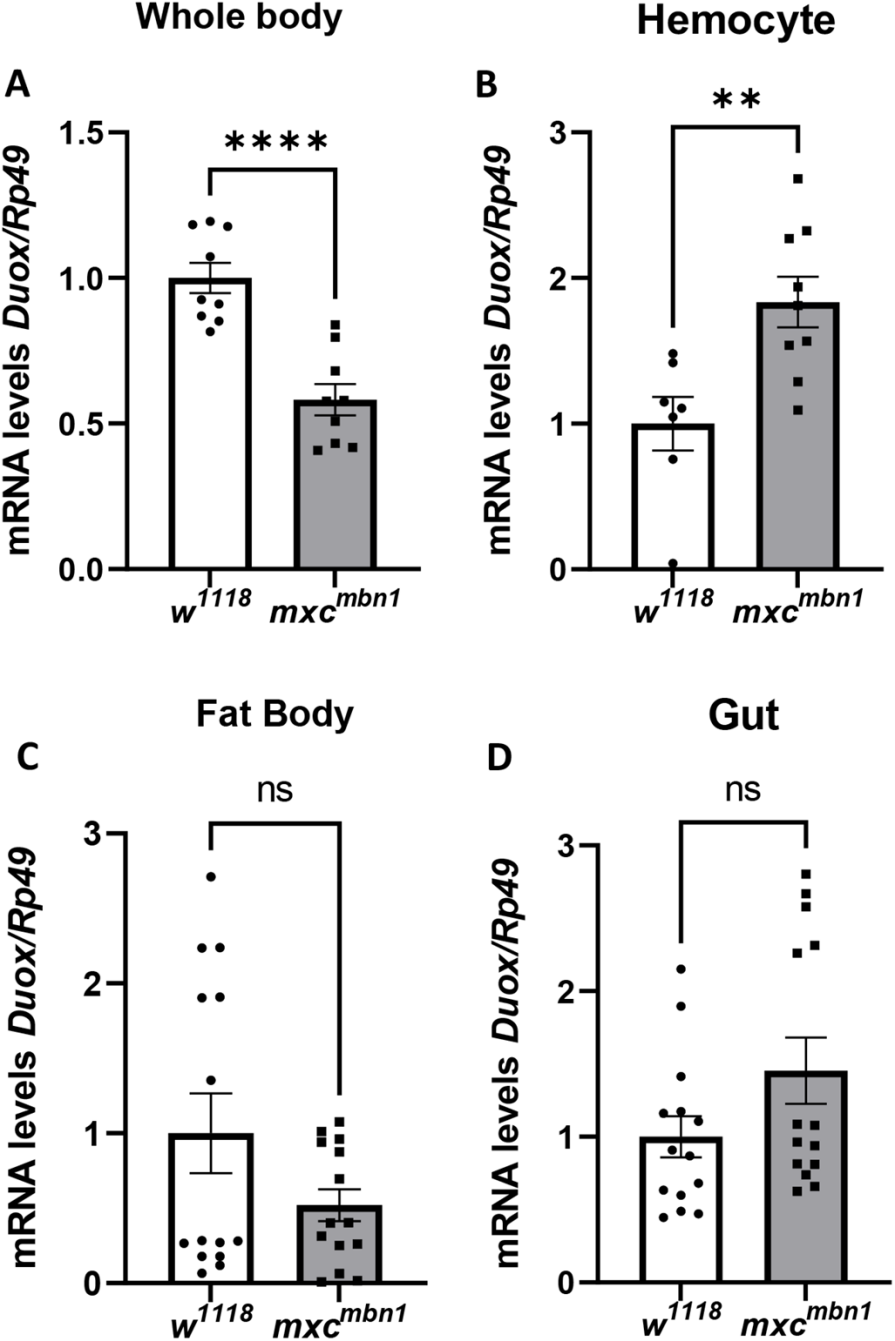
**Increased levels of the *Duox* mRNA in circulating hemocytes in hemolymph of *mxc^mbn1^*.** (A-D) Quantification of *Duox* mRNA by qRT-PCR using total RNAs prepared from whole third instar larvae at mature stage (A, *n*=3), from hemocytes of the third instar larvae (B, *n*=3), from fat bodies (C, *n*=5), and gut (D, *n*=5) as templates. A relative mRNA levels of *Duox* to an internal control (*Rp49*) was calculated. The average level in normal control (*w^1118^*) is presented as 1.0. The *Duox* mRNA levels are represented in the order of the control (white), and *mxc^mbn1^* (gray). The average level in normal control (*w^1118^*) is presented as 1.0. Error bar: s.e.m.

To identify the genes involved in ROS accumulation, we investigated whether the mRNA level of a Dual Oxidase gene (*Duox*) changed in the mutant larvae. We quantified the mRNA level of *Duox* in the whole larvae, circulating hemocytes in the hemolymph, fat body, and gut of control (*w^1118^/Y*) and *mxc^mbn1^* larvae at the third instar stage (Fig. 3A-D). The average *Duox* mRNA level in whole *mxc^mbn1^* larvae was 58.2%, and that in fat body was 54.8% of that of the control (*P*<0.01) (Fig. 3A,C). In contrast, the level in *mxc^mbn1^* larvae hemocytes was twofold higher than that in the control (Fig. 3B); the level in gut of *mxc^mbn1^* larvae hemocytes was also 50% higher than that in the control (Fig. 3D). Thus, *Duox* expression was stimulated in *mxc^mbn1^* larvae, particularly in hemocytes and gut.

**Fig. 4.**
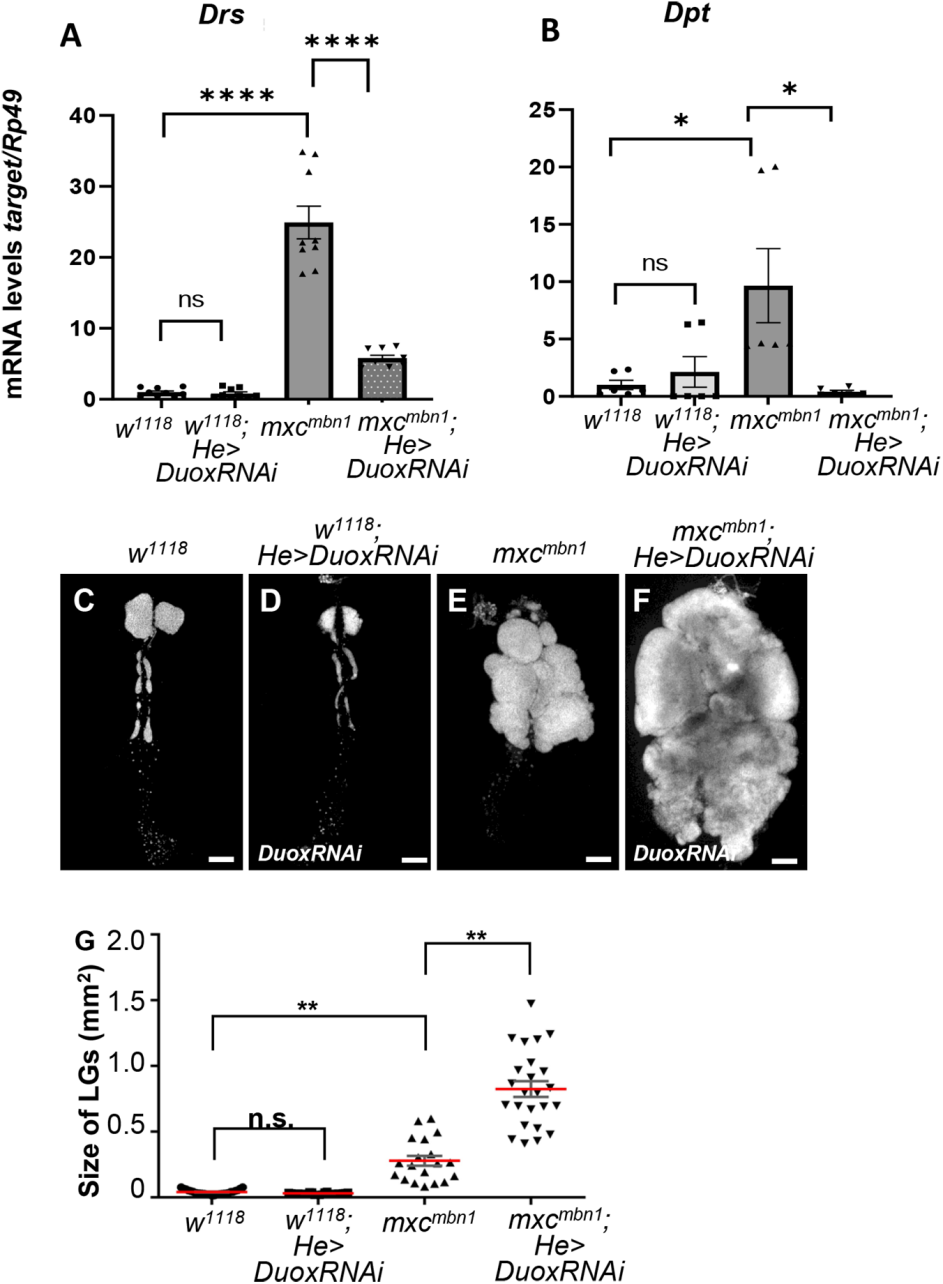
**Reduced mRNA levels of two AMP genes and increased size of the LGs in third instar larvae harboring hemocyte-specific depletion of the *Duox*.** (A,B) Average mRNA levels of *Drs* (A) and *Dpt* (B) in fat bodies of third instar larvae at mature stage by qRT-PCR. Total RNA prepared from fat bodies of normal control larvae (*w^1118^/Y*), *mxc^mbn1^* larvae (*mxc^mbn1^/Y*), *mxc^mbn1^* larvae harboring hemocyte-specific expression of *Duox* (*mxc^mbn1^/Y; He>DuoxRNAi*) was used as templates. The average level in normal control (*w^1118^*) is presented as 1.0. Error bar: s.e.m. (C-F) The DAPI-stained LGs from matured third instar larvae of control (*w^1118^/Y*) (C), control larvae harboring hemocyte-specific depletion of *Duox* (*w^1118^ /Y; He>DuoxRNAi*) (D), *mxc^mbn1^* larvae (*mxc^mbn1^/Y*) (E), *mxc^mbn1^* larvae harboring hemocyte-specific depletion of *Duox* (*mxc^mbn1^/Y; He>DuoxRNAi*) (F). Scale bar: 200 µm. (G) Average size of the LGs (*n*≥19). For statistical analysis of the differences in the LG size, one-way ANOVA with Scheffe's multiple comparison test was performed. Red lines indicate average size of LGs. Error bars: s.e.m.

Next, we investigated whether the upregulation of *Duox* in *mxc^mbn1^* hemocytes was required for activation of innate signaling in the fat body, and thereby suppression of LG hyperplasia. After *Duox* was efficiently depleted using dsRNA against its mRNA (Fig. S3), we confirmed by DHE staining that the Duox depletion in hemocytes resulted in reduced ROS accumulation in the hemocytes (Fig. S4). Then, we performed qRT-PCR to quantify the mRNA levels of *Drs* and *Dpt* in fat body of the mutant larvae with or without hemocyte-specific depletion of *Duox* (Fig. 4A,B). In *mxc^mbn1^* larvae harboring hemocyte-specific *Duox* depletion, the mRNA levels of *Drs* and *Dpt* significantly decreased to 35.2% and 13.3% compared to the levels in the mutant larvae without the depletion, respectively (Fig. 4A,B). These results indicated that *Duox* expression and the resultant ROS accumulation in the circulating hemocytes were required for the activation of the innate signaling pathways in *mxc^mbn1^* larvae*.*

Hence, we next investigated whether *Duox* depletion resulted in enhancement of the LG hyperplasia in the *mxc^mbn1^* larvae. In *mxc^mbn1^* larvae harboring hemocyte-specific depletion of *Duox* (*mxc^mbn1^/Y; He>DuoxRNAi*), the average LG size (0.82 mm^2^, *n*=24) was significantly larger than that of *mxc^mbn1^* without the depletion (0.28 mm^2^, *n*=19) (*P<*0.01) (Fig. 4C-G). As a result, the mutant larvae harboring the Duox depletion (*mxc^mbn1^/Y; He>DuoxRNAi*) contained two times more circulating hemocytes (average 1306.45±409.36 cells/µl hemolymph, *n*=6 larvae), derived from the LG than the *mxc^mbn1^* larvae (*mxc^mbn1^/Y; He>+*) (average 2935.74±652.44 cells/µl hemolymph, *n*=7 larvae). Collectively, *Duox* expression in hemocytes was required for activation of the innate signaling pathway and suppression of LG hyperplasia in *mxc^mbn1^* larvae.

**Fig. 5.**
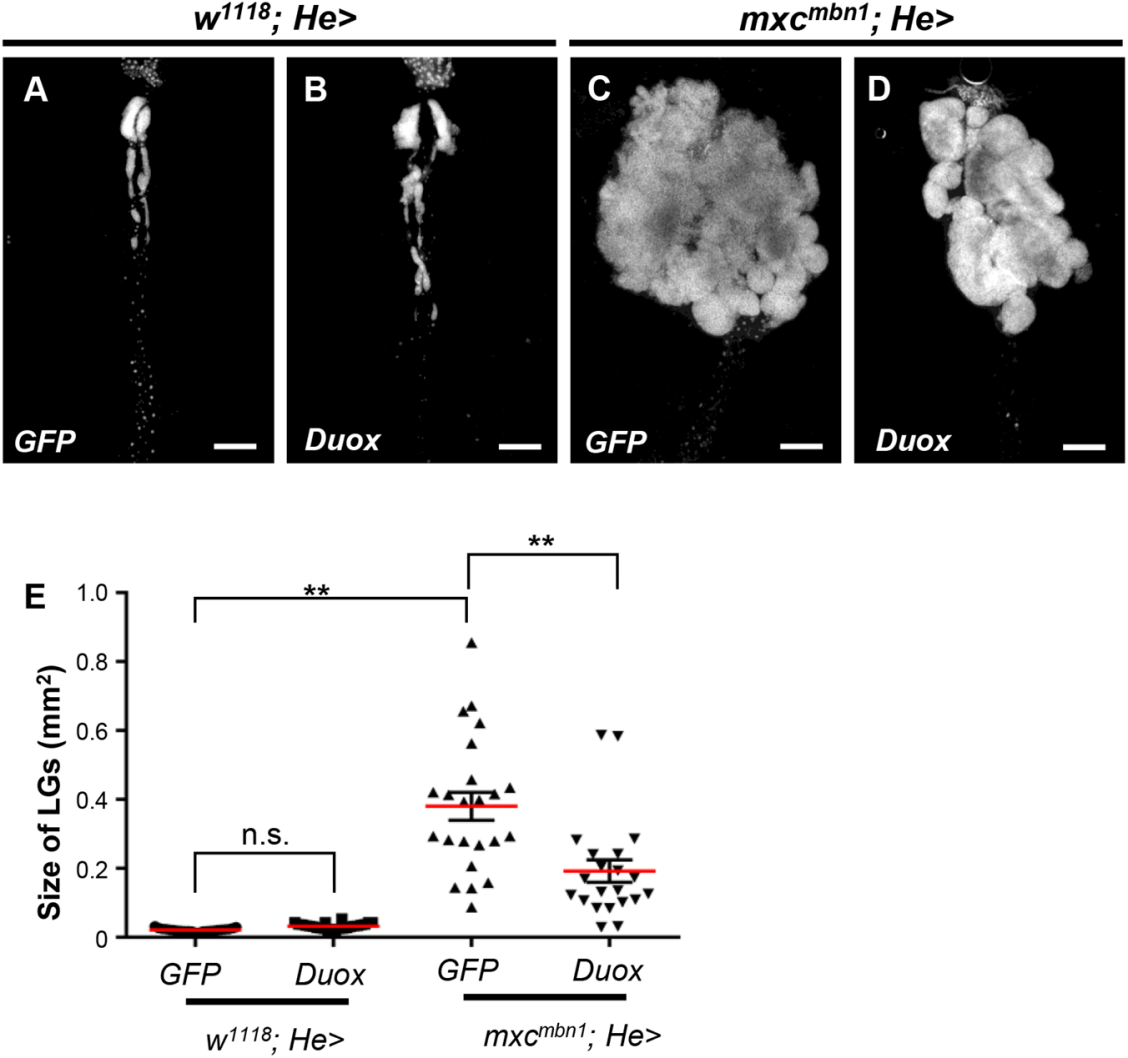
**Reduced size of the tumorous LGs in in *mxc^mbn1^* larvae harboring hemocyte-specific overexpression of *Duox* in *mxc^mbn1^*.** (A-D) The DAPI-stained LGs from mature third instar larvae of control male (*w/Y; He>GFP*) (A), and control larvae harboring hemocyte-specific overexpression of *Duox* (*w/Y; He>Duox*) (B), *mxc^mbn1^* (*mxc^mbn1^/Y; He>GFP*) (C), and the mutant larvae harboring hemocyte-specific overexpression of *Duox* (*mxc^mbn1^/Y; He>Duox*) (D). Scale bar: 200 µm. (E) A quantification of the LGs in *mxc^mbn1^* larvae harboring hemocyte-specific overexpression of *Duox.* For statistical analysis of the differences of the LG size between in *mxc^mbn1^* larvae and in the mutant harboring hemocyte-specific overexpression, one-way ANOVA with Scheffe's multiple comparison test was performed (*n*>20). Red lines and error bars represent average value and s.e.m., respectively.

Furthermore, we investigated whether LG hyperplasia was suppressed by ectopic expression of *Duox* in the hemocytes (Fig. 5A-D). The average LG size (0.19 mm^2^, *n*=21) in *mxc^mbn1^* larvae with hemocyte-specific expression of *Duox* (*mxc^mbn1^/Y; He>Duox*) was significantly smaller than that in *mxc^mbn1^* expressing GFP (*mxc^mbn1^/Y; He>GFP*) (0.39 mm^2^, *n*=25) (*P<*0.01) (Fig. 5E), indicating that ectopic expression of the ROS-producing enzyme resulted in suppression of LG hyperplasia in *mxc^mbn1^* larvae. Consequently, the mutant larvae harboring the *Duox* over-expression in mature hemocytes (*mxc^mbn1^/Y; He>Duox*) contained less circulating hemocytes (average 922.51±242.17 cells/µl hemolymph, *n*=5 larvae), derived from the LG than the *mxc^mbn1^* larvae (*mxc^mbn1^/Y; He>+*) (average 1306.46±409.36 cells/µl hemocytes, *n*=7 larvae). In summary, *Duox* expressed in circulating hemocytes and the resultant ROS in the cells played an essential role in suppression of LG hyperplasia.

### Downregulation of a maturation factor for Duox in the hemocytes also inhibited the innate signaling pathway and enhanced LG hyperplasia in *mxc^mbn1^* larvae

Numb-interacting protein (NIP), encoded by *moladietz* (*mol*), is one of known regulatory factors of Duox in *Drosophila* (Xie et al., 2010). We investigated whether *mol* mRNA level increased in hemocytes of *mxc^mbn1^* larvae using a *mol-lacZ* reporter. Weak anti-β-galactosidase immunostaining signal was detected in the control hemocytes (*w^1118^/Y; mol-lacZ/+*) (Fig. S5A′). In contrast, a stronger signal was observed in the mutant cells (Fig. S5B′). *mol* expression was significantly higher in *mxc^mbn1^* hemocytes (median 541.43, *n*=742) than in control cells (median 472.58, *n*=1148) (*P<*0.0001) (Fig. S5C). Thus, *mol* expression was enhanced in the hemocytes of *mxc^mbn1^* larvae. The mRNA level of the gene in the mutant larvae was five times higher than the level in control larvae at the same developmental stage (Fig. S6D). Thus, *mol* was upregulated in the LG tumor mutant larvae.

Next, we investigated whether the upregulation of the gene in hemocytes was required for activation of the innate immune pathway in the mutant fat body. We performed qRT-PCR to quantify the mRNA levels of *Drs* and *Dpt* using total RNAs prepared from fat body of *mxc^mbn1^* larvae harboring *mol* depletion in the hemocytes (*mxc^mbn1^/Y; He>molRNAi*) (Fig. S6A). The mRNA level of *Drs*, but not that of *Dpt*, was reduced by 46.3% in *mxc^mbn1^* larvae harboring hemocyte-specific depletion of *mol*, compared to that in the mutant larvae without the depletion (Fig. S6B). These results suggested that activation of Toll-mediated innate immune pathway in the fat body was suppressed by hemocyte-specific depletion of the Duox activator.

Next, we investigated whether *mol* depletion in the hemocytes affected LG hyperplasia in the *mxc^mbn1^* larvae. Significant enhancement of LG hyperplasia was observed in *mxc^mbn1^* larvae harboring the depletion (*mxc^mbn1^/Y; He>molRNAi*) (average LG size was 0.25 mm^2^, *n*=24), compared to the average size (0.19 mm^2^, *n*=22) in *mxc^mbn1^* larvae with control depletion (*P*<0.01) (*mxc^mbn1^/Y; He>GFPRNAi*) (Fig. S6C-G). Collectively, upregulation of the maturation factor of *Duox* also activated the Toll-mediated innate signaling pathway to suppress LG hyperplasia. These results are consistent with the findings that Duox in hemocytes was required for activation of the innate immune pathway in the *mxc^mbn1^* fat body.

### Circulating hemocytes transplanted from normal larvae were associated with the LG tumors in *mxc^mbn1^* larvae

**Fig. 6.**
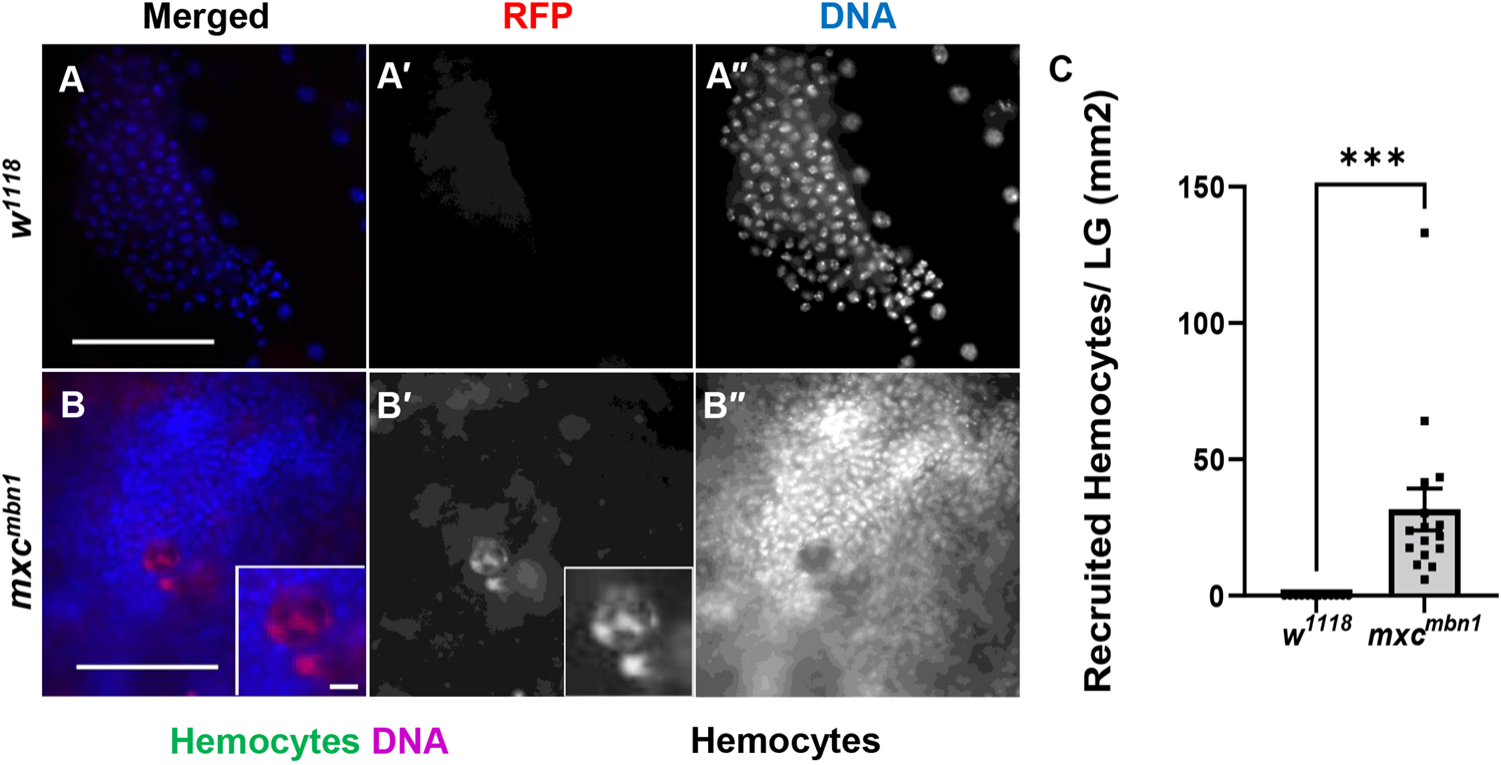
**Circulating hemocytes from normal larvae were associated with the LG tumors in *mxc^mbn1^* larvae.** (A,B) Fluorescence image of DAPI-stained LGs from third instar larvae at mature stage, in which RFP-labelled normal hemocytes were transplanted. (A) Normal control LG consisting of the first and second lobes (*w^1118^/Y*). (B) The most anterior region of the LG, corresponding to one-fifth of the whole LG in the *mxc^mbn1^* larva (*mxc^mbn1^/Y*). (Inset in B) Higher magnification image showing hemocytes on LG. The hemocyte nuclei stained with DAPI (B′) were localized on a focal plane that differed from that in which the fat body nuclei were localized, suggesting that the cells were attached to the surface of the LG. RFP fluorescence, red; DAPI staining, blue. Scale bar: 100 μm. (C) Quantification of RFP-positive hemocytes observed on the LG. The numbers of hemocytes on each LG were converted to the number of cells per unit area of the tissue (mm^2^). The Mann–Whitney *U* test was performed for statistical analysis of the differences in the LG size (12 control and 16 *mxc^mbn1^* LGs were examined).

A previous study has reported that hemocytes containing AMPs secreted from the fat body were attached to the LG tumors in *mxc^mbn1^* larvae (Araki et al., 2019). Therefore, we hypothesized that circulating hemocytes recognized the LG tumors in *mxc^mbn1^* larvae. To confirm this, we investigated whether the hemocytes localized to the mutant LGs*.* As it was difficult to distinguish the circulating hemocytes and LG cells, we investigated whether normal hemocytes transplanted from normal larvae were recruited to the LG tumors. We collected 0.8 μl hemolymph containing normal hemocytes expressing RFP from control larvae (*w; He>RFP*) and transplanted them into dorsal abdomens of control (*w^1118^/Y*) and *mxc^mbn1^* larvae at the third instar stage (*mxc^mbn1^/Y*). Twenty hours after the transplantation, we did not observe any RFP^+^ hemocytes on the LGs in normal larvae after transplanting same volume of the normal hemolymph (*n*=12). By contrast, 25.5 RFP^+^ cells (median) were scored on the *mxc^mbn1^* LG, when the numbers of the hemocytes observed were converted to them per unit area of the tissue (mm^2^) (Fig. 6B,C), whereas (Fig. 6A,C). These genetic data indicated that normal circulating hemocytes can be recruited to the LG tumors in *mxc^mbn1^* larvae.

### Upregulation of *Mmp* genes in *mxc^mbn1^* larvae

**Fig. 7.**
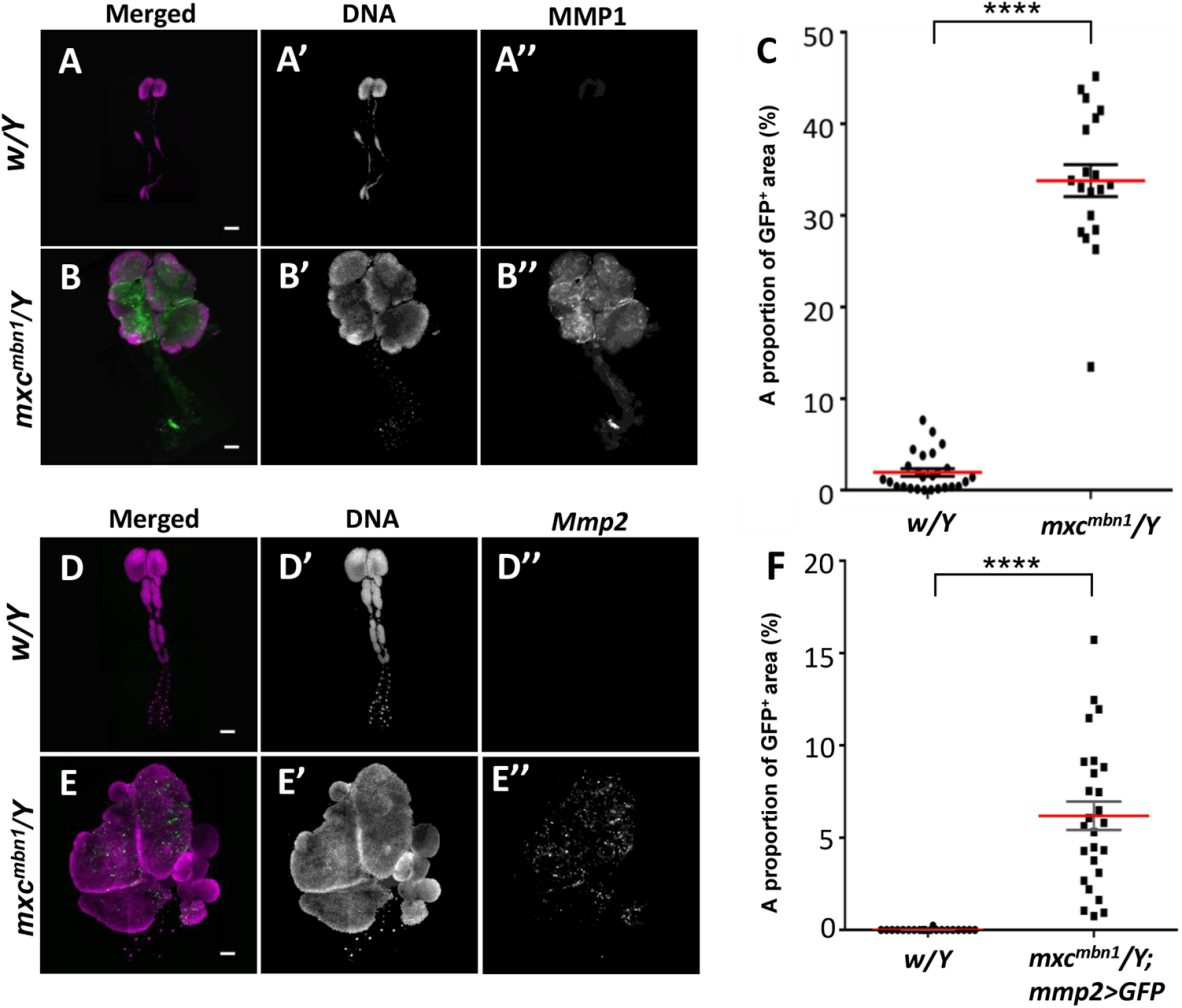
**A higher expression of two Matrix metalloproteinases (MMPs) in LGs of *mxc^mbn1^* larvae.** (A,B) Anti-MMP1immunostaining of whole LGs from third instar larvae; (A) normal control larva (*w^1118^/Y*), and (B) *mxc^mbn1^* larva (*mxc^mbn1^/Y*). Anti-MMPI immunostaining: green (white in A′, B′). DAPI staining, magenta (white in A″, B″). Scale bar: 100 μm. (C) The proportion of the MMP1-positive region in the LGs from third instar larvae. A percentage of each area per a LG hemisphere was plotted in the graph. For statistical analysis of the differences in the LG size, Mann–Whitney U test was performed (*n*>20). (D,E) A visualization of the LG area showing GFP expression under the *Mmp2* enhancer in normal control (*w^1118^/Y; Mmp2>GFP*) (D), and *mxc^mbn1^*(*mxc^mbn1^/Y; Mmp2>GFP*) (E). Scale bar: 200 μm. GFP fluorescence: green (white in D′, E′). DAPI staining, magenta. (F) The proportion of the region showing *MMP2* gene expression in the LGs from third instar larvae. A percentage of each area per a LG hemisphere was plotted in the graph. For statistical analysis of the differences in the LG size, Mann–Whitney *U* test was performed (*n*≥20). Red line and error bar represent median value and s.e.m., respectively.

Next, we addressed the mechanism via which the hyperplasic LG tumor were recognized by hemocytes. For that, we investigated whether there are abnormalities in the tissue integrity of the LG tumors. In the other *Drosophila* tumor mutants, ECM in the hypertrophic imaginal discs was decomposed by MMP (Diwanji and Bergmann, 2020; Pastor-Pareja et al., 2008). We hypothesized that MMP2 was induced in the *mxc* tumorous LGs, where, they decomposed ECM, and that the hemocytes that recognized the matrix pieces assembled onto the dysplastic sites. To verify this, we examined whether MMP genes were expressed in the *mxc^mbn1^* LG. *Drosophila* expresses MMP1 and MMP2. Anti-MMP1 immunostaining revealed that the protein was expressed in the cortical zone (CZ) of the LG (Fig. 7A,A″). As the homogenous immunostaining signal was not observed in all cells of the *mxc* tumor LGs, we measured the MMP1-positive regions of the tumor LGs (Fig. 7B,B″,C). The region was restricted to 8.0% (median, *n*=24) of the entire region in control LG. In contrast, the proportion of the regions increased to 26.2% (median, *n*=28) and it increased significantly in the *mxc^mbn1^* tumor LGs (Fig. 7C) (*P<*0.0001).

We next examined whether MMP2 was expressed in the *mxc^mbn1^* LGs. As antibodies against *Drosophila* MMP2 are not available, we observed GFP fluorescence in control and *mxc^mbn1^* LGs harboring *Mmp2-Gal4*, which expressed *Gal4* under the *Mmp2* enhancer. We did not observe any GFP fluorescence in the control LGs (*w/Y;Mmp2>GFP*) (Fig. 7D″), whereas intense fluorescence was observed in the most anterior lobes of the LGs, corresponding to 6.2% of the entire LG region (Fig. 7E″,F). These observations indicated that the mutant LG tumors displayed the overexpression of MMP2, in spite of no detectable basal level expression in the control LGs.

### Reduced distribution of basement membrane component in the LGs overexpressing *Mmp*2 in *mxc^mbn1^* larvae

**Fig. 8.**
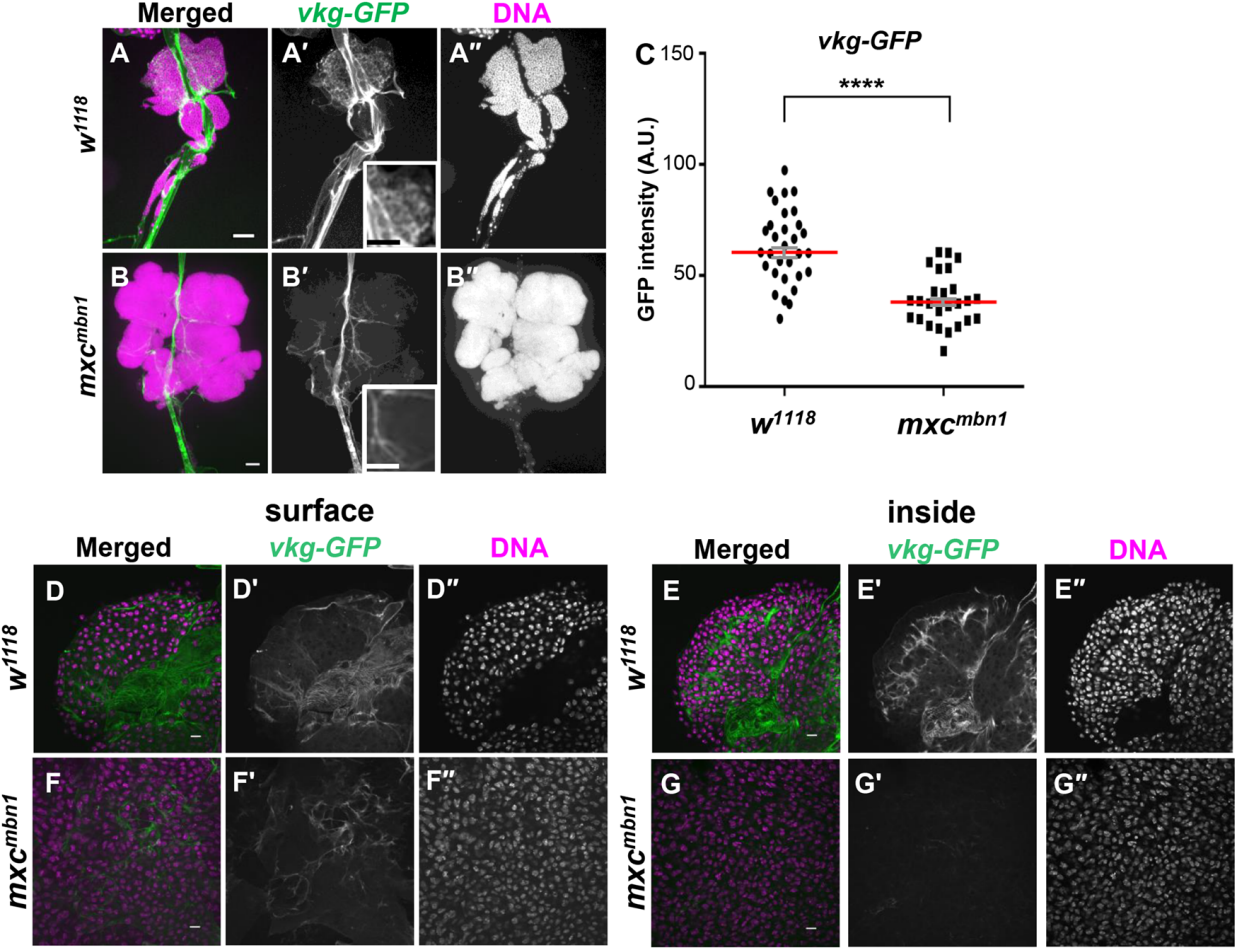
**Distribution of the basement membrane component, collagen IV, in wholemount LG from normal and *mxc^mbn1^* larvae.** (A,B) Conventional fluorescence micrographs of GFP-tagged collagen IV in each whole LG arranged bilaterally, flanking the dorsal vessel, isolated from third instar control larva (*w/Y; vkg-GFP/+*) (A) and *mxc^mbn1^* larva (B) (*mxc^mbn1^/Y; vkg-GFP/+*). (C) Average fluorescence intensity of GFP per lobes of a LG hemisphere from the control and the mutant larvae (arbitrary unit). For statistical analysis of differences in the LG size, the Mann–Whitney *U* test was performed (*n*≥27). Red lines represent median values of the GFP intensity. Scale bars: 100 µm. (D-G) Confocal micrographs of GFP-Collagen IV around LG cells on the surface (C,E) and those inside of the wholemount LGs (F,G) from control (*w/Y; vkg-GFP/+*) (D,F) and *mxc^mbn1^* larvae (*mxc^mbn1^/Y; vkg-GFP/+*) (E,G). Magenta: DAPI staining; green: GFP fluorescence.

We showed that the overexpression of *Mmp2* in the *mxc* tumor LG was sufficient to activate the innate immune pathways. To investigate whether the integrity of basement membrane structure was perturbed in the *mxc* tumor LGs, we initially visualized the whole LGs from normal third instar larvae harboring *vkg-GFP* (Figs. 8A,B) under a conventional fluorescence microscope. The collagen IV signal was densely distributed inside the most anterior lobes of the normal LGs (Figs 8A, 8A′, and inset of 8A′). Intense signals were also observed along the dorsal vessel. In contrast, considerably fewer signals, some of which ran along the outer periphery of the hyperplasic lobes, were observed in the *mxc* tumor LG (Fig. 8B,B′, and inset of 8B′), although the strong signal in dorsal vessels did not change. Next, we monitored the basement membrane from the surface to the inside of the LGs using a confocal microscope. The GFP signal was abundant in both the surface (Fig. 8D) and on the inside of the confocal plane at approximately 15 µm depth from the surface of the control LGs (Fig. 8E). In contrast, we observed a decline in the fluorescence intensity in the LGs of *mxc^mbn1^* larvae from surface to the depth. To minimize the loss of fluorescence from the inside of thicker LG tumors, the LGs from control and the mutant larvae were mildly squashed after fixation to prepare the tissue samples of a similar thickness. The intensity remarkably decreased further and eventually we failed to detect any fluorescence signal at the middle of the LG (Fig. 8G), although the signals were prominent on the surface (Fig. 8F). Furthermore, we observed the LG samples from the control and the mutant larvae under a conventional fluorescence microscopy and quantified the average fluorescence intensity of Vkg-GFP per the lobe areas of the LG hemispheres (Fig. 8C). Significant reduction in GFP fluorescence was observed in the *mxc* tumor LGs, compared to normal controls (*n*>20, *P*<0.0001). Collectively, we concluded that the basement membrane was disassembled or lost in the *mxc* tumor LG consistent with the higher expression of Mmp2.

### Depletion of *Mmp2*, rather than that of *Mmp1*, reduced *Drs* induction in *mxc^mbn1^* larvae

**Fig. 9.**
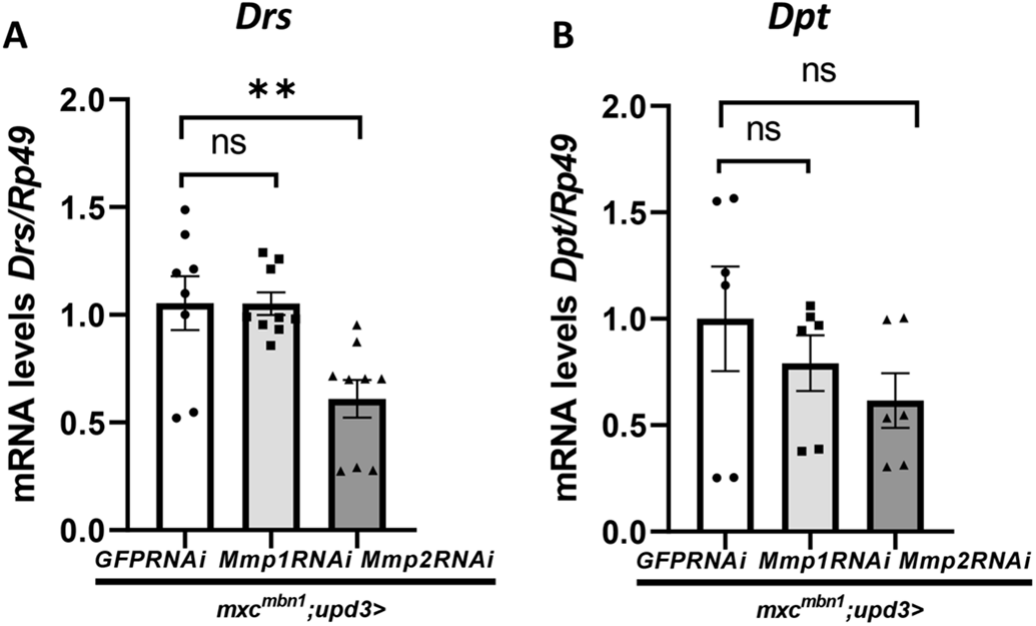
**Altered mRNA levels of two genes encoding Drs and Dpt in the fat body of *mxc^mbn1^* larvae harboring LG cell-specific depletion of MMP1 and MMP2.** (A,B) Relative mRNA levels of two AMP genes, *Drs* (A) and *Dpt* (B) in fat body. Open bars: qRT-PCR using total RNA from fat body harboring control depletion using dsRNA against GFP mRNA in the immature cells of *mxc^mbn1^* LGs (*mxc^mbn1^/Y; upd3>GFPRNAi*). Light gray bars: qRT-PCR using total RNA from fat body of *mxc^mbn1^* larvae harboring the *Mmp1* depletion in the LGs (*mxc^mbn1^/Y; upd3>Mmp1RNAi*). Dark gray bars: qRT-PCR using total RNA from fat body of *mxc^mbn1^* larvae harboring Mmp2 depletion in the LGs (*mxc^mbn1^/Y; upd3>Mmp2RNAi*). The average level in *mxc^mbn1^/Y; upd3>GFPRNAi* is presented as 1.0. Error bar: s.e.m.

Finally, we investigated how the overexpression of *Mmp1* or *Mmp2* influenced AMP expression in the fat body. Firstly, we confirmed that dsRNAs against *Mmp1* and *Mmp2* efficiently depleted the relevant mRNAs in the LG tumor cells (Fig. S7A,B). Then, we performed qRT-PCR experiments using total RNAs prepared from the fat body of *mxc^mbn1^* larvae harboring control depletion (*mxc^mbn1^/Y; upd3>GFPRNAi*), and *Mpm1* (*mxc^mbn1^/Y; upd3>Mmp1RNAi^1^* or *Mmp1RNAi^2^*) or *Mpm2* depletion (*mxc^mbn1^/Y; upd3>Mmp2RNAi*) in the LGs. The levels of *Drs* and *Dpt* mRNAs did not change significantly after the *Mmp1* depletion in the *mxc^mbn1^* LGs using either *UAS-Mmp1RNAi* stock (middle, Fig. 9A,B). In contrast, the levels of *Drs* and *Dpt* in *mxc^mbn1^* larvae harboring the *Mmp2* depletion in LGs commonly decreased by 60.9% and 61.6% of the levels in the mutant LG with control depletion (left, Fig. 9A,B). The difference in mRNA levels of *Drs*, but not *Dpt*, was statistically significant. Thus, downregulation of *Mmp2*, rather than *Mmp1*, influenced activation of the innate immune pathway in the fat body.

## DISCUSSION

### Disassembly of basement membrane in the LG tumors overexpressing *Mmp2* was involved in tumor recognition in *mxc^mbn1^* larvae

MMPs function as indispensable regulators of cell–cell interactions by controlling the ECM turnover (Page-McCaw et al., 2007). In many human tumors, MMP genes are known to be upregulated. This evidence suggested that these proteinases are closely related to tumor growth and progression (Egeblad and Werb, 2002). *Drosophila* cells express two types of MMPs, MMP1 and MMP2, during late larval to pupal stages (Page-McCaw et al., 2003). MMP1 preferentially cleaves DE-cadherin to disrupt cell adhesion, for example in the fat body (Jia et al., 2014). In contrast, MMP2 disassembles fat body cells by cleaving basement membrane components (Jia et al., 2014). This is advantageous for the invasion of the tumor cells generated in *Drosophila* imaginal discs (Srivastava et al., 2007; Uhlirova and Bohmann, 2006). In this study, we showed that both MMP1 and MMP2 are highly expressed in the hyperplasic LGs in *mxc^mbn1^* larvae. We demonstrated that MMP2, rather than MMP1 is required for the activation of the innate signaling pathway in response to the LG tumors in the mutant larvae, although we cannot exclude the possibility that the amount of *Mmp1* mRNA left was enough to trigger the AMP induction, while the *Mmp2* amount was not. Indeed, signals of the basement membrane components were reduced or lost in the *mxc* tumor LGs. This is because higher expression of *Mmp2* enhanced the decomposition of the basement membrane in the mutant larvae, as demonstrated. Furthermore, we observed that the hemocytes were recruited on the LGs in *mxc^mbn1^* larvae (Araki et al., 2019; this study). Another study also reported that the hemocytes were associated with the imaginal disc regions, in which the basement membranes were damaged by JNK-mediated MMP2 from overgrown tissue in *Drosophila* (Diwanji and Bergmann, 2020). Our study further demonstrated that overexpression of *Mmp2*, rather than *Mmp1* contributes to the activation of the innate immunity pathways in the fat body of *mxc^mbn1^* larvae. This finding also corroborates the observation of a previous report that MMP2 drives hemocyte recruitment to overgrown imaginal discs (Diwanji and Bergmann, 2020). Based on the data, we speculated that the hemocytes recognized the loss of basement membrane integrity in the LG cells, and this possibly contributed to activate the innate immune system in the mutant larvae. Moreover, we observed the disassembly of collagen IV inside the LGs, but not on the surface. This suggests that the overexpression of *Mmp2* occurs predominantly in the undifferentiated cells in the medulla zone, rather than in the matured hemocytes in the cortical zone of the LGs. However, we cannot exclude the possibility that the amount of mRNA left in the case of MMP1 was enough to trigger AMPs induction, while MMP2 amount was not. Although we presented evidence suggesting that MMPs are involved in the recognition of the LG tumors by hemocytes, we cannot distinguish it from other possibilities that the MMPs are important for ROS accumulation in the hemocytes and/or the tumor growth.

### Circulating hemocytes producing ROS contribute to the signaling that links *mxc* LG tumor to expression of AMPs in the fat body

More normal circulating hemocytes were associated with the LG tumors than those with the control LGs. We also demonstrated that the overexpression of *Mmp2* in the LG tumors was required for the activation of the innate immune pathway in the fat body, which was distant from the tumor-bearing tissues. Consistently, circulating hemocytes can recognize fragments of ECM generated by MMP ectopically expressed in the tumors in mammal (Nastase et al., 2012), and *Drosophila* (Parisi et al., 2014; Diwanji and Bergmann, 2020; this study). Therefore, we hypothesize that the hemocytes that recognized the tumors stimulated ROS production and might convey the information regarding the existence of the tumors toward the innate immunity-responsible tissues. The results of several previous studies support this hypothesis. For example, circulating hemocytes are required to transmit information regarding bacterial infection or local tissue disintegration toward the fat body (Bosch et al., 2019; Parisi et al., 2014; Pastor-Pareja et al., 2008). Another tumor study in *Drosophila* also reported that expression of *spätzle*, encoding a ligand for Toll, was enhanced in hemocytes in *dlg* mutant larvae harboring tumors. After proteolysis, the active ligand generated could bind and activate the Toll receptor in the fat body (Parisi et al., 2014). These data support our current hypothesis that basement membrane residues of the tumors produced by MMP2 are recognized by hemocytes, which eventually activate innate immune pathway in *mxc^mbn1^* larvae. The mechanism via which the information regarding existence of LG tumors is conveyed toward fat body remains to be studied.

### ROS generated by active Duox in circulating hemocytes may contribute to the activation of the innate immune system in the fat body to induce AMPs

We observed that ROS accumulated in the circulating hemocytes in *mxc^mbn1^* larvae and, further, demonstrated that elimination of ROS by antioxidants suppressed the activation of Toll- and Imd-mediated innate immune signaling pathways in the fat body of *mxc^mbn1^* larvae. Although ROS are known to activate innate immune pathways, it is unlikely that ROS themselves act as signal messengers via the hemolymph, as ROS are unstable *in vivo* (Dickinson and Chang, 2011). Instead, we found that the circulating hemocytes were associated with the LG tumors in *mxc^mbn1^* larvae. Therefore, we speculated that the hemocytes that recognized the tumor cells might directly transfer the information toward the fat body. The Toll-mediated pathway was activated via ROS when *Drosophila* larvae were infected by a parasitoid wasp (Louradour et al., 2017). Moreover, the hemocytes that recognized the stimuli activated the Toll-mediated signaling pathway and consequently induced the expression of *Cecropin-C* (*CecC*) (Chakrabarti and Visweswariah, 2020). In mammalian cells, ROS is consistently required for activation of the IKK complex in the innate immune pathway and for stimulating nuclear import of Relish (Morgan and Liu, 2011). These observations suggested that ROS play an important role in activating innate immune signaling. Tissue disintegration can be recognized by hemocytes, and the information is transmitted to the fat body producing AMPs (Capilla et al., 2017). In this study, we showed that *Duox* was upregulated in *mxc^mbn1^* larvae. This was indispensable for the activation of the innate immune pathway. Therefore, ROS production and accumulation in circulating hemocytes is required for the activation of the innate immune pathways. Moreover, we demonstrated that a maturation factor for Duox was similarly upregulated in the mutant hemocytes. This supported the idea that the oxidase was critical for the activation of the innate immune pathway. Consistently, its mammalian orthologue, DUOXA, is also essential for translocating the oxidase to the cell surface. DUOXA ensures efficient ROS production by DUOX (Grasberger and Refetoff, 2006; Qin et al., 2004).

Duox and its possible activator, were upregulated in the hemocytes of *mxc^mbn1^* larvae, but not remarkably in the fat body producing AMPs. This is consistent with a model that the hemocytes conveyed the ROS toward the fat body. ROS is involved in the proteolysis that generates the active ligand, Spätzle, which binds to the Toll receptor (Louradour et al., 2017). In larvae harboring epithelial tumors, Spätzle is also induced and activated via ROS-dependent proteolysis in hemocytes, which subsequently activates the Toll receptor (Parisi et al., 2014). Therefore, it is important to confirm that the expression of *Spätzle* and ROS-dependent activation might be enhanced in the hemocytes in the mutant larvae. Further investigations are warranted to be performed to propose more reliable model.

Considering our results and those of previous studies, we proposed the following model to explain the mechanism via which *Drosophila* LG tumors are detected and eliminated by the innate immune system. Overexpressed MMP2 in the LG tumors in *mxc^mbn1^* larvae degrades the basement membrane in the tissue. The hemocytes that recognize the tissue disintegration accumulated on the LG tumors. Consequently, simultaneous induction of *Duox*, they produced the oxidase and secreted ROS around the cells. Thereby, the hemocytes producing ROS continuously can convey the information regarding the existence of tumors toward the fat body. Thereafter, the cells may migrate to the fat body and activate the Toll-mediated signaling pathway on the tissue using active Toll ligand generated via ROS-dependent proteolysis (Louradour et al., 2017). Finally, the AMPs were produced and secreted from the fat body to suppress the LG tumor growth.

In this study, we observed that the disassembly of basement membrane due to the overexpressed MMP2 was involved in the tumor recognition by macrophage-like hemocytes in *mxc^mbn1^* larvae in *Drosophila*. Although several studies have demonstrated the ectopic expression of MMPs in epithelial tumors, only a few of them have presented substantial evidence that the disassembly of basement membrane by MMP2 is required for tumor recognition (Beaucher et al., 2007; Kudo et al., 2012). Our previous study established the association between the hemocytes that incorporated AMPs with tumors; however, the mechanism by which the hemocytes recognized the ECM fragments remained elusive (Araki et al., 2019). These findings allow us to speculate the mechanism by which macrophage-like *Drosophila* hemocytes also relay the information toward the fat body apart from the tumors and activate the innate immune system. In our future studies, we would like to obtain more evidence suggesting the mechanism regarding activation of the Toll ligand on the fat body of *mxc^mbn1^* larvae by the ROS-generating hemocytes.

## MATERIALS AND METHODS

### *Drosophila* stocks and their husbandry

Canton S was used as the wild-type stock, and *w^1^* as the normal control stock. A stock carrying the *mxc* lethal mutation showing the LG tumor phenotype (*mxc^mbn1^*) were used (Araki et al., 2019; Kurihara et al., 2020a,b; Remillieux-Leschelle et al., 2002). As heterozygotes for the mutation were maintained under a balancer chromosome carrying *sqh-RFP*, the hemizygotes were selected as larvae without RFP expression. For the UAS-dependent expression of mRNAs or dsRNA in hemocytes, we used *P*{*w^+mC^=He-Gal4.Z*}(*He-Gal4*) obtained from the Bloomington Drosophila Stock Center (BDSC, Indiana University, Bloomington, IN, USA) (Stofanko et al., 2008). To induce the gene expression in immature hemocyte precursors in lymph gland, *P{upd3-GAL4}*(*upd3-Gal4*) (Jung et al., 2005) from N. Perrimon (Harvard Medical School, Boston, MA, USA) was used.

For the ectopic expression of Duox (dDuox), MMP1 and MMP2, we used *UAS-dDuox* (*UAS-Duox*) (Ha et al., 2005), which was a gift from W. Lee (Seoul National University, Republic of Korea), *P{w^+mC^=UAS-Mmp1.f1}3* (*UAS-Mmp1*) (BL58701 from BDSC) and *P{w^+mC^=UAS-Mmp2.P}* (*UAS-Mmp2*) (BL58706 from BDSC), respectively. For dsRNA-dependent gene silencing of genes encoding Dual oxidase (Duox), *mol* gene for a Numb-interacting protein (NIP), genes for Mmp1, and Mmp2, *P{TRiP.GL00678}attP40* (*UAS-DuoxRNAi^GL00678^*) from BDSC (BL38907 from BDSC), *P{TRiP.HMS02560}attP40* (*UAS-molRNAi^HMS02560^*) (BL42867 from BDSC), *P{TRiP.JF01336}attP2* (*UAS-Mmp1RNAi^2^*) (BL31489 from BDSC), *P{KK108894}VIE-260B* (*UAS-Mmp1RNAi^1^*) from the Vienna Drosophila Resource Center (VDRC) (Vienna, Austria) (#101505), and *P{TRiP.HMJ23143}attP40* (*UAS-Mmp2RNAi*) from National Institute of Genetics (Mishima, Japan) (#11605) were used. To monitor gene expression of the *mol* gene, and *Mmp2* gene, *P{lacW}mol^k11524a^* (*mol-lacZ*) (BL12173 from BDSC) and *P{w^+mW.hs^=GawB}Mmp2^NP0509^* (*Mmp2-GAL4*) (Srivastava et al., 2007) (#103625 from DGRC) were used, respectively. To visualize basement membrane component, collagen IV, *PBac{fTRG00595.sfGFP-TVPTBF}VK00033* (*Vkg-GF*P)(#318167 from VDRC) was used, respectively. A redox GFP reporter, *P{gstD1-GFP.S}* (*gstD1-GFP*) was used to estimate the extent of oxidative stress accumulation in larval tissues (Sykiotis and Bohmann, 2008; Le et al., 2019). This stock was a gift from D. Bohmann (Rochester University, Rochester, NY, USA).

All *Drosophila* stocks were maintained on standard cornmeal food, as previously described (Oka et al., 2015). Gal4-dependent expression was measured at 28°C. Other experiments and stock maintenance were conducted at 25°C.

### LG preparation

Normal controls (*+*/Y or *w*/Y) pupated at 6 days (28°C) and 7 days (25°C) after egg laying (AEL), whereas some of the *mxc^mbn1^* mutant remained in third instar larval stage at 10 days (28°C) and 11 days (25°C) AEL (Kurihara et al., 2020a,b). To minimize the possibility of a delay that might allow the tissue to grow, the comparative analysis of hemizygous mutants and controls was performed on the same day (5 days AEL at 28°C), when the wandering third instar larval stage was seen. Alternatively, the tissues were collected from hemizygous mutant larvae 1 day after the timing of the LG collection from control larvae. For the staging of the larvae, parent flies were transferred into a new culture vial and left there to lay eggs for 24 h. A pair of anterior lobes of the LG without connected cardiac cells from mature stage larvae were isolated and fixed with 3.7% formaldehyde for 5 min. The fixed samples were mildly flattened under constant pressure using an apparatus so that the tissue became spread out into cell layers with a constant thickness. A pair of lobes of the LG without connected cardiac cells from mature stage larvae were isolated and fixed with 3.7% formaldehyde. The fixed each LG pair was mildly flattened under constant pressure so that the tissue became spread out into cell layers with a constant thickness as described (Araki et al., 2019). The microscope images acquired as multiple images were assembled to single images using photoshop (CS6 version, Adobe systems, San Jose, CA, USA). The LG area of each DAPI-stained sample was measured using ImageJ ver.1.47 (https://imagej.nih.gov/ij/).

### LG immunostaining

For immunostaining, LGs were dissected from matured third instar larvae and fixed in 3.7% paraformaldehyde for 15 min. After repeated washing, the fixed samples were incubated with primary antibody at 4°C for overnight. The following anti-Mmp1 antibodies (#3A6B4, #3B8D12, and #5H7B11) were mixed and used (1:100 for each; DSHB, IA, USA). After extensive washing, specimens were incubated with Alexa 594 secondary antibody (1:400; Molecular Probe, USA). The LG specimens were observed under a fluorescence microscope (Olympus, Tokyo, Japan, model: IX81), outfitted with excitation, emission filter wheels (Olympus). The fluorescence signals were collected using a 10x dry objective lens. Specimens were illuminated with UV filtered and shuttered light using the appropriate filter wheel combinations through a GFP filter cube. GFP fluorescence images were captured with a CCD camera (Hamamatsu Photonics, Shizuoka, Japan). Image acquisition was controlled through the Metamorph software version 7.6 (Molecular Devices, Sunnyvale, CA, USA) and processed with Adobe Photoshop CS. The basement membrane of the LG cells was observed under a confocal microscope from the surface to the inside of the tissue (Fv10i, Olympus, Tokyo, Japan) by altering the focus along the z-axis. The confocal images obtained were then processed by the Fv10i software and Adobe photoshop CS (Adobe KK, Tokyo, Japan).

### Immunostaining of hemocytes in larval hemolymph

Single mature larvae at the third instar stage were dissected in *Drosophila* Ringer solution (DR) (10 mM, pH 7.2, Tris-HCl, 3 mM CaCl_2_・2H_2_O, 182 mM KCL, 46 mM NaCl) on a slide glass so as to allow circulating hemocytes to release into the DR outside the larvae. Whole aliquots of the cell suspension were collected as much as possible, and hemocytes in the cell suspension were counted using a hemocytometer (Araki et al., 2019). The cells were fixed in 4% paraformaldehyde for 10 min after placing the small amount of DR containing circulating hemocytes on a slide glass and leaving for evaporating. We used anti-β-galactosidase antibody (MP Biomedicals (Irvine, CA, USA), #02150039, 1:2000), anti-P1 antibody for plasmatocytes (Kurucz et al., 2007, 1:100), which was a gift from I. Ando (Hungarian Academy of Sciences, Budapest, Hungary) as primary antibodies. The number of circulating hemocytes in the fluorescence microscope images was counted by ImageJ.

For monitoring ROS in hemocytes and larval tissues, dihydroethidium (DHE) (#181094, Life Technologies, Carlsbad, CA, USA) was used. We quantified the intensity levels of the DHE fluorescence in hemocytes, the GFP fluorescence in the hemocytes of *gstD-GFP* larvae, and anti-β-galactosidase immunostaining signal in *mol-lacZ* using ImageJ. The cells were classified into five categories according to the intensity levels: cells showing less than 4000 intensity values in ImageJ were of background level or below category (I), those with 4000 to 20,000 intensity values were in category II, with 20,000 to 40,000 intensity values in moderate category (III), with 40,000 to 60,000 intensity values in intense category (IV), and higher than 60,000 intensity values in the more intense category (V). We classified the GFP-positive cells in *gstD-GFP* larvae into four groups according to the fluorescence intensity; cells showing less than 30 values were in category I, 30 to 70 in category II, 70 to 150 in III, and more than 150 in IV.

### qRT-PCR analysis

Total RNA was extracted from whole larvae at third instar stage, and larval fat body, guts, LGs and hemocytes in hemolymph with each genotype using the TRIzol reagent (Invitrogen, Carlsbad, CA, USA). cDNA synthesis from the total RNA was carried out using the PrimerScript High Fidelity RT-PCR Kit (TaKaRa, Shiga, Japan) using an oligo dT primer. Real-time PCR was performed using the FastStart Essential DNA Green Master (Roche, Mannheim, Germany) and a Light Cycler Nano instrument (Roche, Mannheim, Germany). According to a software the Primer3Plus (http://www.bioinformatics.nl/cgi-bin/prime r3plus.cgi), the following primers were synthesized:

**Table:**
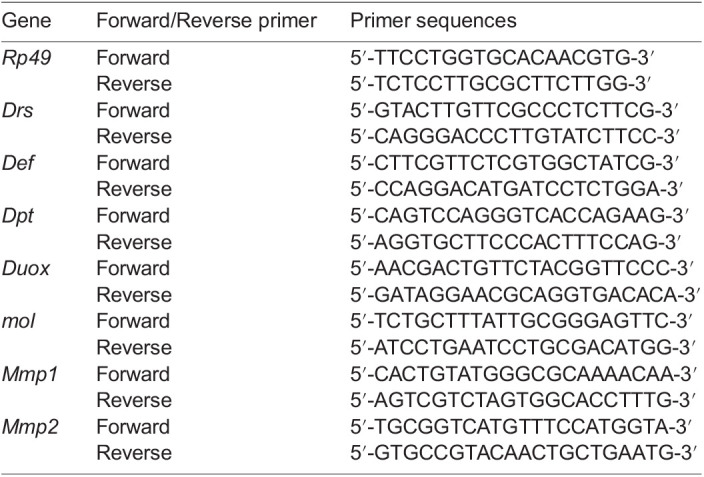


### Transplantation of hemocytes in *Drosophila* larvae

A small aliquot (0.8 μl) of larval hemolymph containing hemocytes (2151±301 cells on average) or pieces of the LG were performed using a glass needle into a recipient third instar larva. The needles were generated from G1.2 (Narishige Co., Tokyo, Japan) using a puller, PN-31, and used after sharpening of the tip. The donor hemocytes were injected within 5 min after dissection of recipient larvae to avoid melanization and clogging. The injected larvae were put on wet blocking papers for 1 h to recover them from the damage and raised on a standard food for 20 h before observation.

### Feeding experiments of the antioxidant

Thirty embryos laid within a day were transferred to a standard food supplemented with 0.1 mg/ml N-acetyl cysteine (NAC) (Sigma-Aldrich, St. Louis, MO, USA, #A7250) and allowed the larvae to feed on the food at 25°C.

### Statistical analysis

Results of the LG area measurements were presented as scatter plots created using GraphPad Prism 6 or Microsoft Office Excel 2016 (Microsoft, Redmond, WA, USA). The area in pixels was calculated and an average value was determined for each LG. Each single dataset was assessed using Welch's *t*-test or Student's *t*-test as described previously (Araki et al., 2019; Kurihara et al., 2020a,b). The *F*-test was performed to determine equal or unequal variance. *P*-values were calculated using Welch's *t*-test of unequal variance if the value was less than 0.05. *P*-values were calculated using the Student's *t*-test of equal variance when the *F*-value was greater than 0.05. The statistical significance is described in each figure legend: *P<0.05, **P<0.01, ***P<0.001, and ****P<0.0001.

## Supplementary Material

10.1242/biolopen.059523_sup1 Supplementary information
