# Supplementary material for: Drosophila hemocytes recognize lymph gland tumors of mxc mutants and activate the innate immune pathway in a reactive oxygen species-dependent manner
Source: Biol Open. 2022 Nov 3;11(11):bio059523. doi: 10.1242/bio.059523 (PMC9641529; doi:10.1242/bio.059523)
Supplement: Supplementary information [file biolopen-11-059523-s1.pdf]

Fig. S1

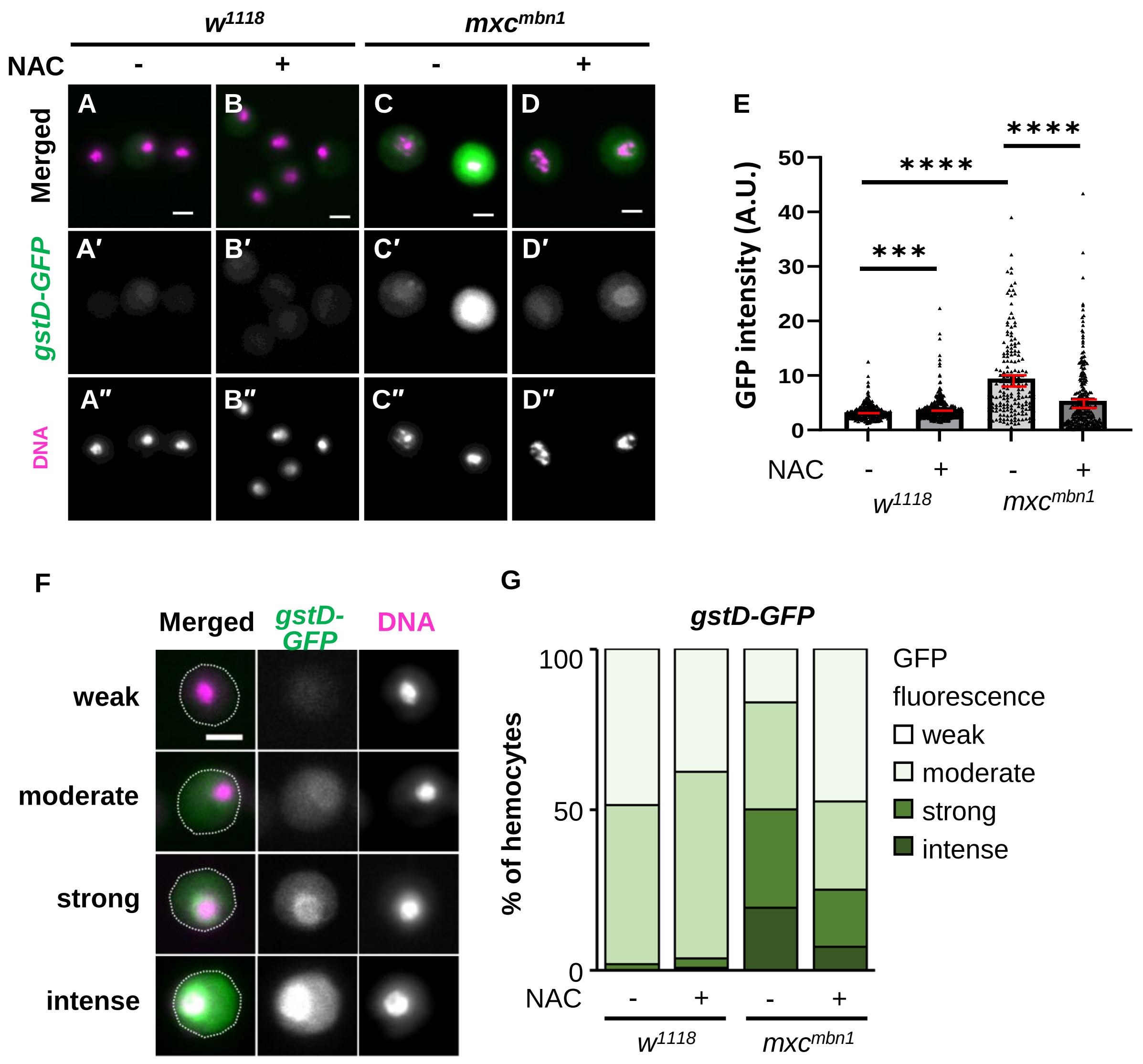

Fig. S1. Reduced expression of a redox reporter, *gstD1-GFP* in circulating haemocytes of *mxc*^{mbn1} larvae fed on N-acetyl Cysteine (NAC).

(A-D) GFP fluorescence of circulating haemocytes prepared from matured 3rd instar larvae carrying the *gstD1-GFP* reporter. (A, C)The hemocytes from a control larva (*w*^{1118}*/Y; gstD-GFP/+*) fed without NAC (A) or fed on NAC(C). (B, D)The cells from the mutant larvae (*mxc*^{mbn1}*/Y; gstD-GFP/+*) fed without NAC (B) or fed on NAC (D).The GFP fluorescence and DNA staining are coloured in green and magenta, respectively.

Bar:5 µm. (E) Distribution of arbitrary units of the GFP fluorescence in circulating hemocytes from control (*w*^{1118}*/Y; gstD-GFP/+*) fed without (n=372 cells) or with NAC (n=466), and *mxc*^{mbn1} mutant larvae (*mxc*^{mbn1}*/Y; gstD-GFP/+*) fed without (n=174) or with NAC (n=258), respectively. The fluorescence intensity of each cell is plotted on the bar. Error bars in red; standard error of mean (s.e.m.). For statistical analysis, Kruskal-Wallis test followed by the Mann-Whitney U test using Bonferroni correction was17 the four classes according to the GFP fluorescence intensity (weak, moderate, strong, intense classes). The cell margins are encircled by dotted lines. Bar: 5 µm. (G) A percentage of each class of the circulating haemocytes showing the GFP fluorescence from control (*w*^{1118}) and *mxc*^{mbn1} larvae fed without NAC or with NAC.

Biology Open • Supplementary information

Fig. S2

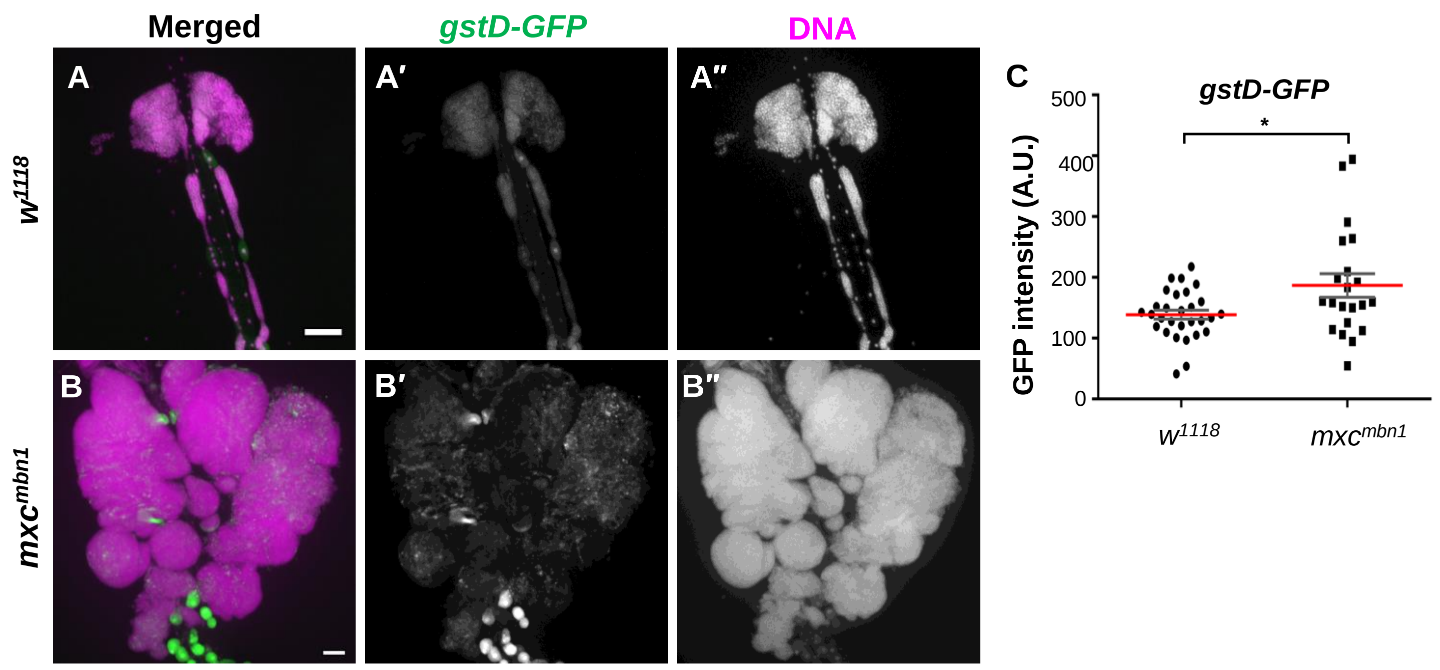

Fig. S2. A slightly higher expression of a redox reporter, *gstD1-GFP* in LGs of *mxc^mbn1* larvae.
(A, B) GFP fluorescence of LGs prepared from matured 3rd instar larvae carrying the *gstD1-GFP* reporter. (A) The LG from a control larva (*w^1118/Y; gstD-GFP/+*), and (B) that from the mutant larvae (*mxc^mbn1/Y; gstD-GFP/+*). The GFP fluorescence (A', B') and DNA staining (A", B") are coloured in green and magenta, respectively. Bar: 100 µm. (C) Average arbitrary units of the GFP fluorescence in whole lobe regions of the LGs (n≥ 21) from control (*w^1118/Y; gstD-GFP/+*) and *mxc^mbn1* mutant larvae (*mxc^mbn1/Y; gstD-GFP/+*). The fluorescence intensity of the LG regions including the hemocyte-looking cells with more intense fluorescence on the mutant LGs, except pericardial cells was measured. Error bars correspond to s.e.m. For statistical analysis, Welch's *t* test was performed (*p<0.05, n≥ 21).

Biology Open • Supplementary information

Fig. S3

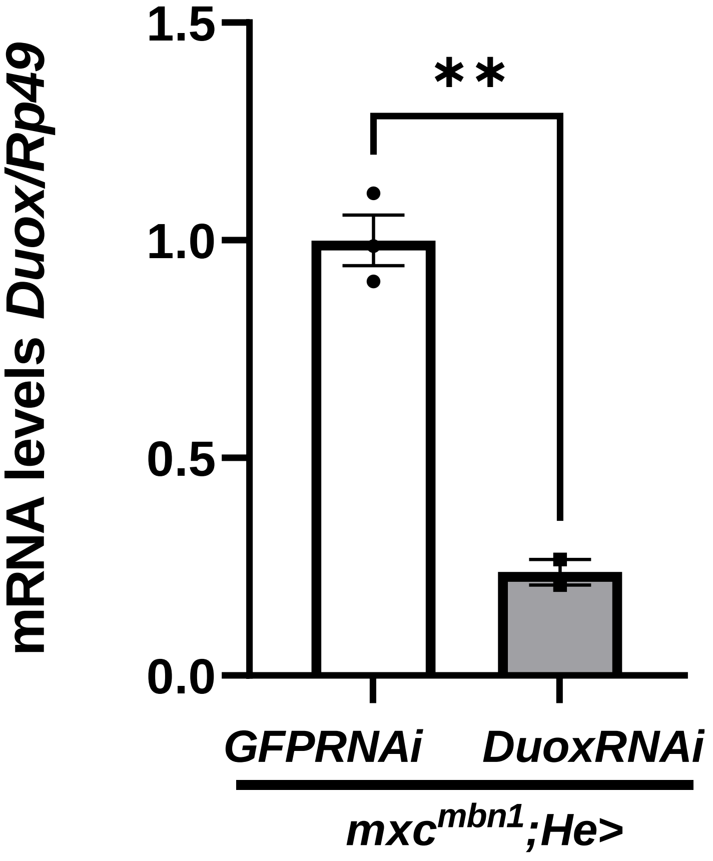

Fig. S3. An efficient depletion of endogenous *Duox* mRNA by induction of dsRNA against its mRNA.

The qRT-PCR experiments was performed using total RNA prepared from circulating haemocytes of the following 3rd instar larvae as templates. The mxc^{mbn1} larvae harbouring hemocyte-specific expression of dsRNA against *GFP* mRNA (mxc^{mbn1}*/Y; He>GFPRNAi*)(left), dsRNA against *Duox* mRNA (mxc^{mbn1}*/Y; He>DuoxRNAi*) (right). Error bar represents standard error of mean (**p<0.01, Welch's t test).

Biology Open • Supplementary information

Fig. S4

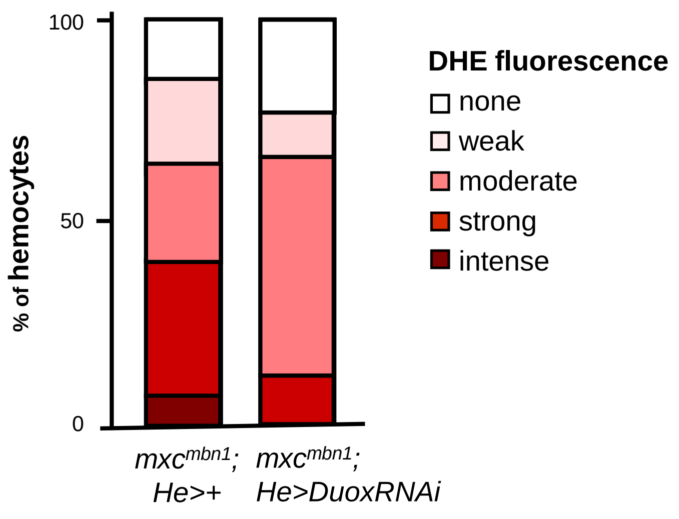

Fig. S4. A classification of the *mxc*[*mbn1*] circulating hemocytes and the mutant hemocytes harbouring hemocyte-specific depletion of *Duox* stained with DHE according to the fluorescence intensity.

The *mxc*[*mbn1*] hemocytes (n = 278) were classified into none fluorescence class (15.8%), weak class (23.7%), moderate class (24.5%), strong class (30.6%), and intense class (5.4%). In contrast, *mxc*[*mbn1*] hemocytes harbouring *Duox*RNAi (n = 286) were classified into none fluorescence class (2.4%), weak class (26.2%), moderate class, (47.9%) and strong class (23.4%), and intense class (0.0%). Note that DHE fluorescence intensity is reduced in the mutant hemocytes harbouring the *Duox* depletion.

Biology Open • Supplementary information

Fig. S5

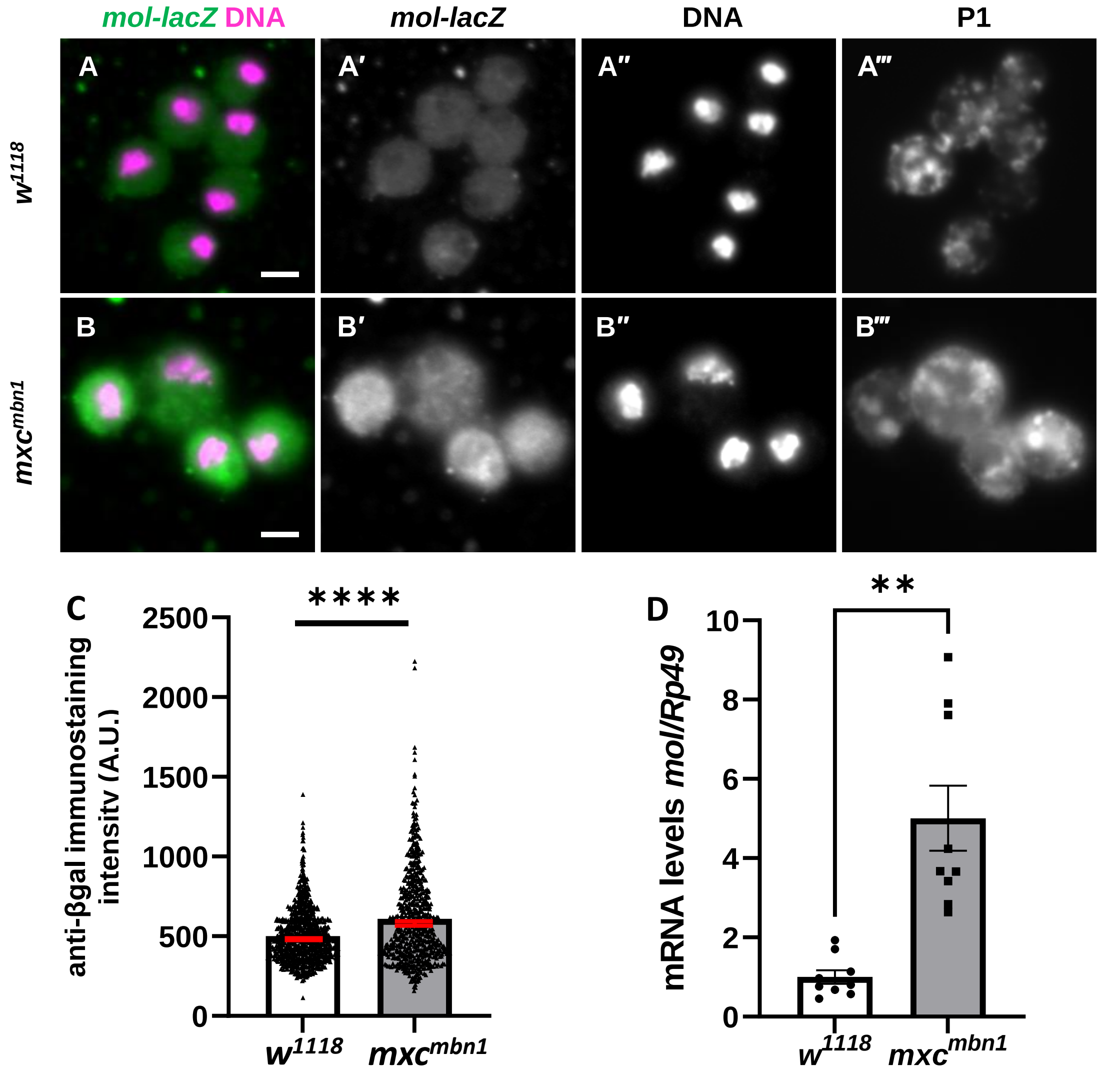

Fig. S5. Increased mRNA levels of *moladietz* (*mol*) gene in circulating haemocytes of *mxc*^{mbn1} larvae.
(A, B) Ant-β-galactosidase immunostaining of circulating haemocytes in 3rd instar stage larvae at mature stage. Circulating haemocytes in a normal larva (*w*^{1118}*/Y; mol-lacZ/+*) (A), in a *mxc*^{mbn1} larva (*mxc*^{mbn1}*/Y; mol-lacZ/+*)(B). Anti β-galactosidase immunostaining staining are coloured in green (white in A′, B′), and DNA staining by DAPI are magenta (white in A″, B″). (A‴, B‴) Anti-PI immunostaining of matured plasmatocytes in larval haemolymph. Bar; 5 μm. (C) A distribution of fluorescence intensity in circulating haemocytes stained by anti-β-galactosidase immunostaining (A.U.). Each intensity is plotted on the bars. Open bar; average fluorescence intensity of normal control haemocytes, gray bar; average fluorescence intensity of *mxc*^{mbn1} haemocytes. Forstatistical analysis of the differences in the LG size between in *mxc*^{mbn1} larvae and in the mutant harbouring haemocyte-specific overexpression, Mann-Whitney U test was performed (****$p<0.0001$, n≥742). Error bars in reds represent s.e.m. (D) Average mRNA levels of circulating haemocytes quantified by qRT-PCR (n=3). mRNA levels of circulating haemocytes in a normal larva (*w*^{1118}*/Y*), and *mxc*^{mbn1}(*mxc*^{mbn1}*/Y*). Error bars; standard error of mean.

Biology Open • Supplementary information

Fig. S6

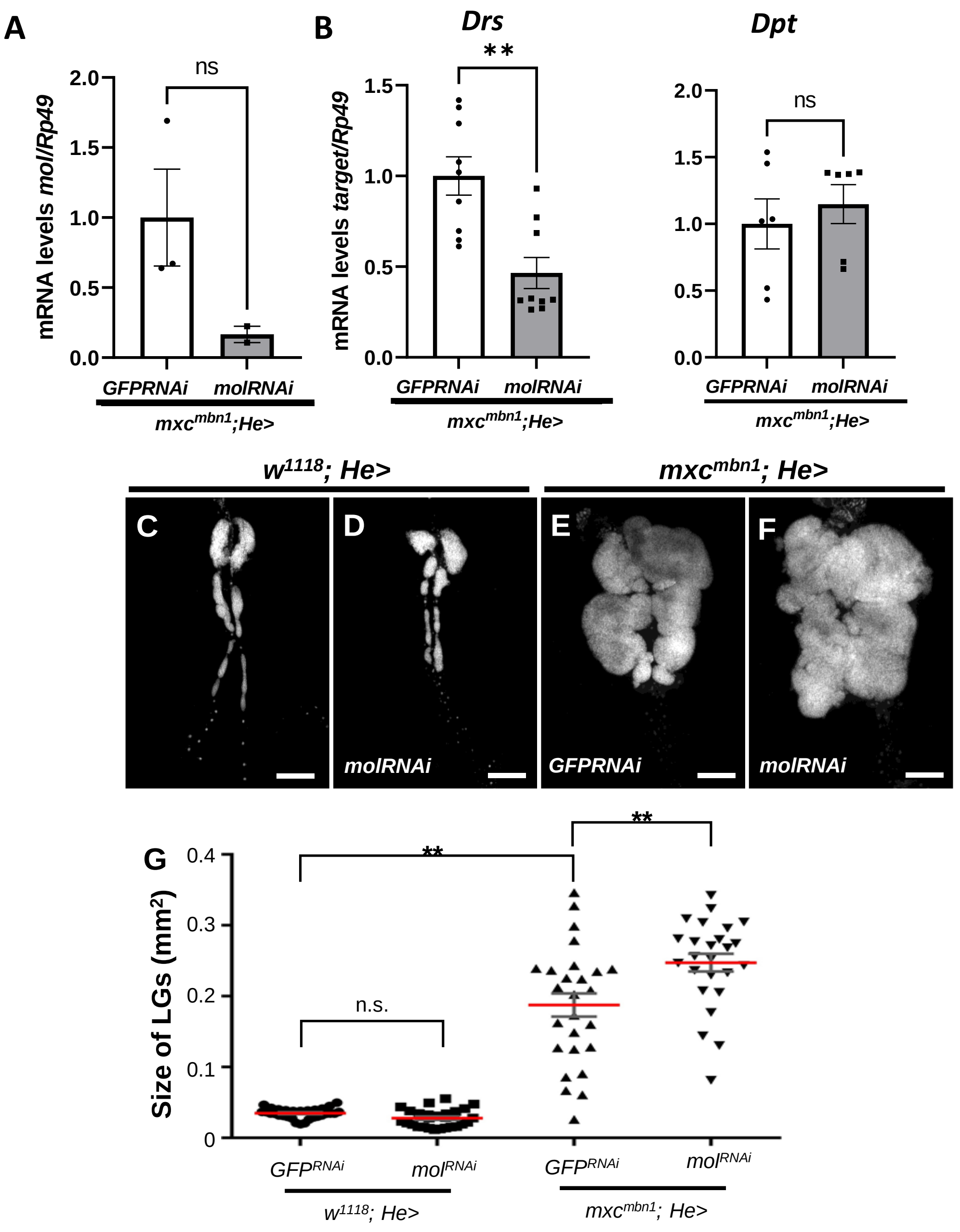

Fig. S6. Reduced levels of *Drs* mRNA and enlarged LG in *mxc*^{mbn1} larva harbouring haemocyte-specific depletion of *mol.*

(A) An efficient depletion of endogenous *mol* mRNA by induction of dsRNA against its mRNA. cDNA produced using total RNA from circulating haemocytes was used for qRT- PCR experiment. Open bar; a relative level of *mol* mRNA in circulating haemocytes harbouring haemocyte-specific expression of dsRNA against *GFP* mRNA (*mxc*^{mbn1}*/Y; He>GFPRNAi*), gray bar; a relative *mol* mRNA level in circulating haemocytes harbouring haemocyte-specific depletion of *mol* mRNA in *mxc*^{mbn1} larvae (*mxc*^{mbn1}*/Y; He>molRNAi*). Error bars; standard error of mean. (B) A quantification of *Drs* and *Dpt* mRNAs in fat bodies by qRT-PCR. Open bars; mRNA levels of two AMP genes in fat bodies in *mxc*^{mbn1} larvae harbouring haemocyte-specific expression of dsRNA against GFP mRNA (*mxc*^{mbn1}*/Y; He>GFPRNAi*), gray bars; the mRNA levels in fat bodies in *mxc*^{mbn1} larvae harbouring haemocyte-specific depletion of *mol* mRNA (*mxc*^{mbn1}*/Y; He> molRNAi*). Error bars; standard error of mean. (C-F) A quantification of the LG size from 3rd instar larvae at mature stage; (C) a control LG harbouring hemocyte-specific expression of dsRNA against *GFP* mRNA (*w*^{1118}*/Y; He>GFPRNAi*), (D) a control LG harbouring haemocyte-specific depletion of *mol* mRNA (*w*^{1118}*/Y; He>molRNAi*), (E) a *mxc*^{mbn1} LG harbouring haemocyte-specific expression of dsRNA against *GFP* mRNA (*w*^{1118}*/Y; He>GFPRNAi*), (*mxc*^{mbn1}*/Y; He>GFPRNAi*), (F) a *mxc*^{mbn1} LG harbouring haemocyte-specific depletion of *mol* mRNA (*mxc*^{mbn1}*/Y; He>molRNAi*). Bar; 200 µm. (G) A quantification of size of the DAPI-stained LGs. For statistical analysis of the differences of the LG size between in *mxc*^{mbn1} larvae and in the mutant harbouring haemocyte-specific overexpression overexpression, one-way ANOVA with Scheffe's multiple comparison test was performed (**$p<0.01$, n.s.: not significant, n>20). Red lines and error bars represent average value and s.e.m., respectively.

Biology Open • Supplementary information

Fig. S7

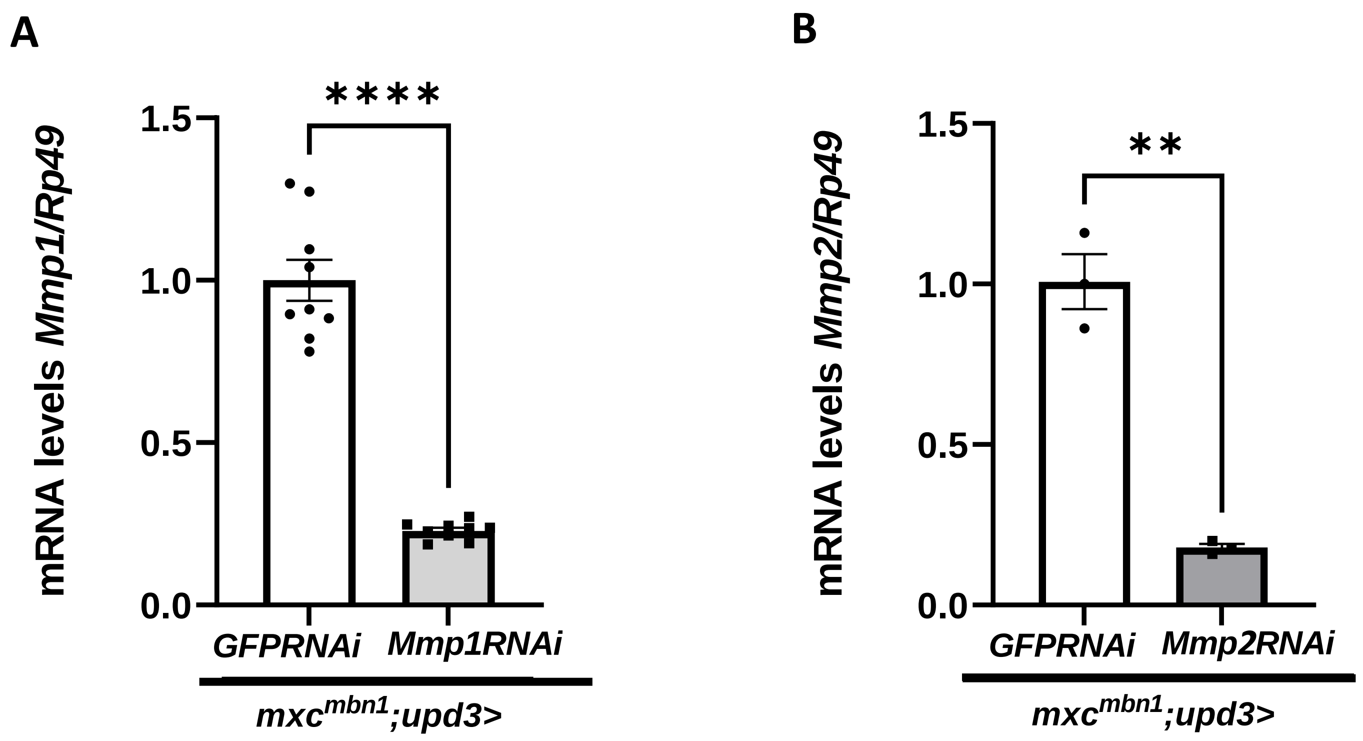

Fig. S7. Efficient depletion of MMP1 and MMP2 mRNAs by the dsRNAs against the relevant mRNAs.

(A) Relative mRNA levels of *Mmp1* by qRT-PCR using total RNAs from the LGs in 3rd instar larvae of *mxc^mbn1* harbouring expression of control dsRNA for GFP mRNA (open bars) (*mxc^mbn1/Y; upd3>GFPRNAi*), and dsRNA against the relevant gene (grey bars) *mxc^mbn1*(*mxc^mbn1/Y; upd3>Mmp1RNAi*) in the immature LG cells, respectively (Welch's *t* test, **p<0.01). (B) Relative mRNA levels of *Mmp2* by qRT-PCR using total RNAs from the LGs in 3rd instar larvae of *mxc^mbn1* harbouring expression of control dsRNA for GFP mRNA (open bars) (*mxc^mbn1/Y; upd3>GFPRNAi*), and dsRNA against the relevant gene (grey bars) *mxc^mbn1*(*mxc^mbn1/Y; upd3>Mmp2RNAi*) in the immature LG cells, respectively. Error bars represents standard error of mean (Welch's t test, *p<0.05).
